# Gene networks associated with conditional fear in mice identified using a systems genetics approach

**DOI:** 10.1186/1752-0509-5-43

**Published:** 2011-03-16

**Authors:** Christopher C Park, Greg D Gale, Simone de Jong, Anatole Ghazalpour, Brian J Bennett, Charles R Farber, Peter Langfelder, Andy Lin, Arshad H Khan, Eleazar Eskin, Steve Horvath, Aldons J Lusis, Roel A Ophoff, Desmond J Smith

**Affiliations:** 1 Department of Molecular and Medical Pharmacology, David Geffen School of Medicine, University of California, Los Angeles, CA 90095, USA; 2 Department of Medical Genetics and Rudolf Magnus Institute of Neuroscience, UMC Utrecht, 3584 CG, Utrecht, The Netherlands; 3 Department of Medicine - Cardiology, David Geffen School of Medicine, University of California, Los Angeles, CA 90095, USA; 4 Department of Human Genetics, David Geffen School of Medicine, University of California, Los Angeles, CA 90095, USA; 5 Department of Computer Science, University of California, Los Angeles, CA 90095, USA; 6 University of California, Los Angeles, Center for Neurobehavioral Genetics, David Geffen School of Medicine, CA 90095, USA; 7 Center for Public Health Genomics, School of Medicine, University of Virginia, VA 22908, USA

## Abstract

**Background:**

Our understanding of the genetic basis of learning and memory remains shrouded in mystery. To explore the genetic networks governing the biology of conditional fear, we used a systems genetics approach to analyze a hybrid mouse diversity panel (HMDP) with high mapping resolution.

**Results:**

A total of 27 behavioral quantitative trait loci were mapped with a false discovery rate of 5%. By integrating fear phenotypes, transcript profiling data from hippocampus and striatum and also genotype information, two gene co-expression networks correlated with context-dependent immobility were identified. We prioritized the key markers and genes in these pathways using intramodular connectivity measures and structural equation modeling. Highly connected genes in the context fear modules included *Psmd6*, *Ube2a *and *Usp33*, suggesting an important role for ubiquitination in learning and memory. In addition, we surveyed the architecture of brain transcript regulation and demonstrated preservation of gene co-expression modules in hippocampus and striatum, while also highlighting important differences. *Rps15a, Kif3a, Stard7, 6330503K22RIK*, and *Plvap *were among the individual genes whose transcript abundance were strongly associated with fear phenotypes.

**Conclusion:**

Application of our multi-faceted mapping strategy permits an increasingly detailed characterization of the genetic networks underlying behavior.

## Background

Advances in both genetic and behavioral techniques are providing unprecedented opportunities for dissecting the gene networks governing behavior. Through a variety of approaches, promising candidate genes have been identified for a wide collection of clinically relevant traits such as anxiety, conditional fear and spatial memory [1-3]. Intercrosses and backcrosses have been widely used to identify behavior quantitative trait loci (QTLs) in mice, but suffer from poor mapping resolution. More recently, the use of outbred mice has allowed fine mapping of a range of biological [3] and expression traits [4,5]. However, outbred mice are a fleeting resource and must be regenotyped and re-phenotyped for each study.

In spite of many successes, the recent wave of genome-wide association studies paints an increasingly complex picture of genes underlying behavioral traits. The genetic architecture of most behaviors is widely distributed, with collections of independent loci making relatively small contributions to overall trait variability [6,7]. The largely undefined and likely complex contribution of environmental factors to both the etiology and maintenance of behavior represents another formidable obstacle to reliable QTL mapping.

Recent work has achieved superior resolution using panels of inbred mouse lines [8]. Power can be further improved by incorporating recombinant inbred (RI) strains formed by crossing classical inbred strains followed by repeated sibling mating. One such resource is the hybrid mouse diversity panel (HMDP) which combines inbred and RI lines to create a panel of 100 strains with great resolution and statistical power [9]. The HMDP consists of 29 classical inbred strains supplemented with 71 RI strains derived from C57BL/6J crossed with either DBA/2J, A/J or C3H/HeJ. In addition to enhanced resolution, there are other significant advantages to using the HMDP for genetic mapping. Each strain has been genotyped extensively [10], and multiple individuals can be phenotyped for the same trait, reducing measurement variability. Furthermore, the panel is a renewable resource, since each strain can be propagated indefinitely [11]. Phenotype data can be pooled and shared in an ongoing fashion, while the effects of environmental variables are easily studied.

**Figure 1:**
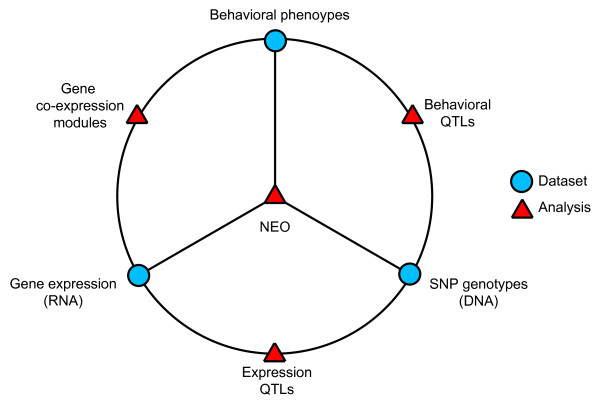
**A systems biology approach to dissecting fear biology**. Data from behavioral phenotype analysis was integrated with SNP genotypes to map behavioral QTLs. Behavioral phenotypes were also compared to gene co-expression modules created from hippocampus and striatum microarray datasets. Gene expression data and SNP genotypes were used together to map expression QTLs. All three datasets were merged to prioritize mapped genes using Network Edge Orienting. This approach identifies gene networks associated with behavioral phenotypes.

To leverage these emerging resources, we employed an integrative systems approach to explore the genetics of conditional fear. Figure 1 illustrates the sources of data we collect and how we investigate relationships to identify genetic pathways implicated in the predisposition to fear. Mice were phenotyped on a fear conditioning assay, and the quantitative data combined with single nucleotide polymorphism (SNP) genotypes to map behavioral quantitative trait loci (QTLs). We corrected for the confounding effects of relatedness and population structure between strains using efficient mixed model association (EMMA) [12]. By combining genome-wide expression QTL (eQTL) maps for hippocampus and striatum, weighted gene correlation network analysis (WGCNA) [13,14], and structural equation modeling, we identified single genes and pathways with relationships to fear-driven behavioral phenotypes.

## Results

To identify regions of the genome associated with fear-related behavior, mice from the HMDP were subjected to a fear conditioning procedure and characterized on 48 unique behavioral phenotypes drawn from different test phases. Using these phenotypes as quantitative traits, we performed a genome-wide association study (GWAS) to identify loci associated with each of the behavioral traits.

### Cued and context fear phenotyping

Mice were tested for cued and contextual fear acquired through a Pavlovian conditioning procedure. Such fear memories manifest across a variety of behavioral dimensions and can be collectively quantified through the use of automated tracking and analysis [15].

**Figure 2:**
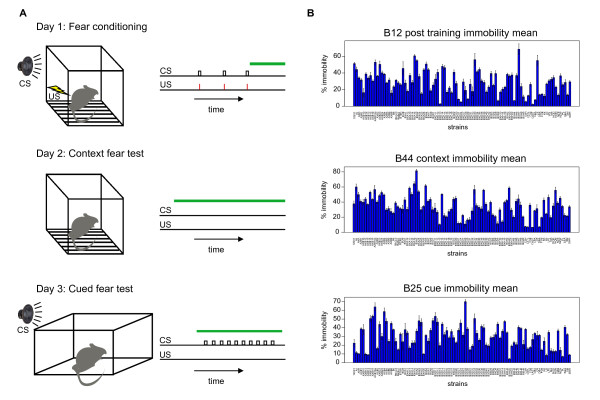
**Fear conditioning in the HMDP**. **A) **Behavioral procedure for cued fear conditioning. Mice were subjected to a three-phase procedure. On day 1, mice received 3 auditory conditional stimuli (CS) co-terminating with 0.75 mA foot shock. On day 2, mice were returned to the conditioning chamber for an 8 minute extinction test. On day 3, mice were placed in a novel chamber and given a series of 10 CS presentations (inter-trial interval 1 minute). Green horizontal lines show time periods when fear endpoints were measured. **B) **Behavioral distributions for selected endpoints across HMDP, corresponding to panels in **A**. Percent immobility calculated for three separate test phases.

Immobility (freezing) is a classical measure of fear triggered by an environmental threat. This species-specific defense response can be reliably acquired in a single conditioning trial, making it a widely used model for fear expression and learning and memory. We also monitored other measures of fear including velocity, thigmotaxis (wall-preference), path shape, and habituation. The fear conditioning assay is depicted schematically in Figure 2A. On day one, a mouse is placed in a cage where an auditory conditional stimulus (CS) tone is played for fifteen seconds followed by a brief foot shock. Training consisted of three tone-shock pairings. The next day, the mouse returned to the same chamber and contextual fear is indexed through a collection of behavioral endpoints including immobility. On the third day, the mouse is placed in a novel chamber and given a series of CS presentations with no foot shock. Cued fear is quantified across the same behavioral endpoints used to assess contextual fear.

Variability in freezing across the panel is shown in Figure 2B. Further testing details for each of the behavioral phenotypes (labeled from B1 to B48) are provided in Additional file 1 (Supplementary methods and Table S1). A cluster dendrogram depicting the similarity between the quantitative behavioral phenotypes across the HMDP is shown in Additional file 1 Figure S1. Surprisingly, context and cue immobility measures clustered closely together although they index different types of learning.

### Mapping of conditional fear QTLs

We mapped loci for behavioral phenotypes using EMMA and 101,629 SNPs ([12], **METHODS**). Across 48 measured behavioral phenotypes, QTL analysis revealed 27 loci with a *P *value < 4.48 × 10^-6^, corresponding to a genome-wide false discovery rate (FDR) of 5%. This threshold value is comparable to that from another study using the same panel [9], which employed permutation testing to calculate a genome-wide significance threshold of *P *= 4.1 × 10^-6 ^or a family-wise error rate of 0.05. QTL plots for the entire battery of behavioral endpoints are in Additional file 1 Figure S2. The significant loci and corresponding closest genes are summarized in Table 1.

**Table 1:** Behavioral QTLs with FDR < 0.05

Quantitative Behavioral Phenotype	Chromosome	Base Position	Nearest gene	*P *value
B3 pre training thigmotaxis mean distance to point	9	61,060,175	*Tle3*	1.14 × 10^-6^
B6 post training velocity mean	15	5,887,595	*Dab2*	2.92 × 10^-6^
B11 pre training immobility mean	2	6,186,281	*Echdc3*	1.77 × 10^-6^
B11 pre training immobility mean	7	126,370,751	*Gpr139*	1.31 × 10^-6^
B12 post training immobility mean	8	68,297,006	*March1*	4.41 × 10^-6^
B24 precue immobility mean	7	94,641,553	*Tyr*	5.58 × 10^-9^
B24 precue immobility mean	7	94,744,373	*Grm5*	5.58 × 10^-9^
B24 precue immobility mean	7	107,177,259	*Chrdl2*	5.14 × 10^-8^
B25 cue immobility mean	3	103,364,188	*Syt6*	1.56 × 10^-6^
B25 cue immobility mean	3	130,123,970	*Col25a1*	3.44 × 10^-6^
B25 cue immobility mean	4	6,678,672	*Tox*	2.58 × 10^-6^
B25 cue immobility mean	7	94,641,553	*Tyr*	4.40 × 10^-9^
B25 cue immobility mean	7	94,744,373	*Grm5*	4.40 × 10^-9^
B25 cue immobility mean	7	104,540,350	*Alg8*	7.06 × 10^-9^
B25 cue immobility mean	15	37,521,578	*Ncald*	1.76 × 10^-6^
B25 cue immobility mean	19	26,658,546	*Smarca2*	3.80 × 10^-6^
B27 precue mobility mean	7	94,641,553	*Tyr*	1.37 × 10^-6^
B27 precue mobility mean	7	94,744,373	*Grm5*	1.37 × 10^-6^
B30 precue thigmotaxis mean distance to point	1	163,397,742	*Tnfsf18*	3.17 × 10^-6^
B31 cue thigmotaxis mean distance to point	11	48,065,799	*Gnb2l1*	1.24 × 10^-8^
B33 precue thigmotaxis mean	2	151,612,920	*Psmf1*	3.36 × 10^-6^
B33 precue thigmotaxis mean	11	52,523,068	*Fstl4*	2.20 × 10^-6^
B33 precue thigmotaxis mean	13	72,750,827	*D430050G20*	3.73 × 10^-6^
B38 context thigmotaxis mean distance to point	1	172,955,973	*Fcgr4*	1.22 × 10^-6^
B38 context thigmotaxis mean distance to point	8	53,062,087	*Aga*	3.62 × 10^-6^
B38 context thigmotaxis mean distance to point	9	61,070,635	*Tle3*	2.16 × 10^-6^
B42 context meander mean	2	129,472,283	*Sirpa*	3.65 × 10^-6^
B44 context immobility mean	2	128,198,673	*Gm14005*	3.32 × 10^-6^
B44 context immobility mean	6	71,209,634	*Smyd1*	5.22 × 10^-8^
B47 context mobility extinction	11	70,800,475	*Dhx33*	4.27 × 10^-6^

We mapped a highly significant QTL on chromosome 7 for cued immobility (*P *= 4.40 × 10^-9^). There are two peak markers for this locus, located ~102 kb apart and residing in different linkage disequilibrium blocks (Additional file 1 Figure S3). One peak marker is located within the Tyrosinase (*Tyr*) gene. Since the HMDP is composed of inbred mouse strains, a number are homozygous for a recessive mutation in *Tyr *leading to an albino coat color (26 of 94 strains phenotyped).

One study looked directly at the effects of *Tyr *on cue dependent freezing behavior [16] using both B6 mice with a mutant *Tyr *allele and an AJ congenic strain with the wildtype B6 allele substituted for the albino *Tyr *allele. *Tyr *had only a small influence on fear learning with only minor (if any) learning deficits due to reduced visual acuity [17-19] and was one of likely many alleles influencing this phenotype. Interestingly, the second peak has the same *P *value as the first and lies in the glutamate receptor gene metabotropic 5 (*Grm5*), which is involved in glutamatergic neurotransmission. Homozygous null mice for *Grm5 *have been shown to have reduced hippocampal long term potentiation (LTP) [20] and impaired spatial learning [21]. These mice also have a behavioral phenotype associated with a rodent model of schizophrenia [22]. Polymorphism at this locus may contribute to a variance in motor activity as a conditioned response to a tone.

### eQTL mapping in hippocampus and striatum

Using gene expression measures of 25,697 transcripts as quantitative traits from tissue from both the hippocampus (98 strains, *n *= 1) and striatum (96 strains, *n *= 1), we mapped expression quantitative trait loci (eQTLs) and their corresponding expression SNPs (eSNPs) using EMMA ([12], see **METHODS**). For each tissue, we calculated an independent genome-wide significance threshold corresponding to a false discovery rate (FDR or *Q *value) < 5% [23]. In hippocampus, this threshold was *P *< 9.21 × 10^-6 ^while in striatum the corresponding threshold was *P *< 1.19 × 10^-5^. We separated the eSNPs from each tissue into two separate categories: markers within 2 Mb of the probe start position (termed *cis *or local) and markers more than 2 Mb away (termed *trans *or distant).

In hippocampus, we mapped 2,128 *cis *eQTLs, while in striatum we mapped 2,528. There was strong overlap in the *cis *eQTLs of the two tissues with 1,641 in common (*χ*^2 ^= 11,831, *df *= 1, *P *< 10^-300^) indicating that transcription regulation due to polymorphism is strongly preserved between tissues. Interestingly, the set of *cis *eQTLs unique to hippocampus was enriched in genes from the gene ontology (GO) category [24] involved in the "positive regulation of behavior" (*Q *= 1.8 × 10^-3^). The top 100 *cis *eQTLs in each tissue along with locations of their corresponding peak markers and minimum *P *values are provided in Additional file 1 (Tables S2 and S3).

The presence of a SNP within the 50mer probe sequence of the transcripts interrogated by the microarray might produce spurious false positive *cis *eQTLs due to a change in binding avidity. To investigate this possibility, we downloaded a list of 8,265,759 known SNPs from the Perlegen SNP Database http://mouse.cs.ucla.edu/mousehapmap and searched for each of these SNPs in the 25,697 probes on the Illumina microarray. Of the SNPs in this list, 3,841 probes contained at least one SNP. In the hippocampus, we observed 535 eQTLs with SNPs while 317 were expected proportionally (χ^2 ^= 22.0, *df *= 1, *P *< 2.7 × 10^-6^). The striatum also showed slight enrichment with 602 *cis *eQTLs exhibiting SNPs in probes with 372 expected (χ^2 ^= 3.0, *df *= 1, *P *= 0.08). Although probe SNPs did increase the number of observed *cis *eQTLs, the proportion was <15%, suggesting that >85% of *cis *eQTLs do not have evidence of being artifacts due to polymorphism. Of course, other naturally occurring polymorphisms likely exist that are not contained in the Perlegen SNP database and could also lead to false positive associations.

**Figure 3:**
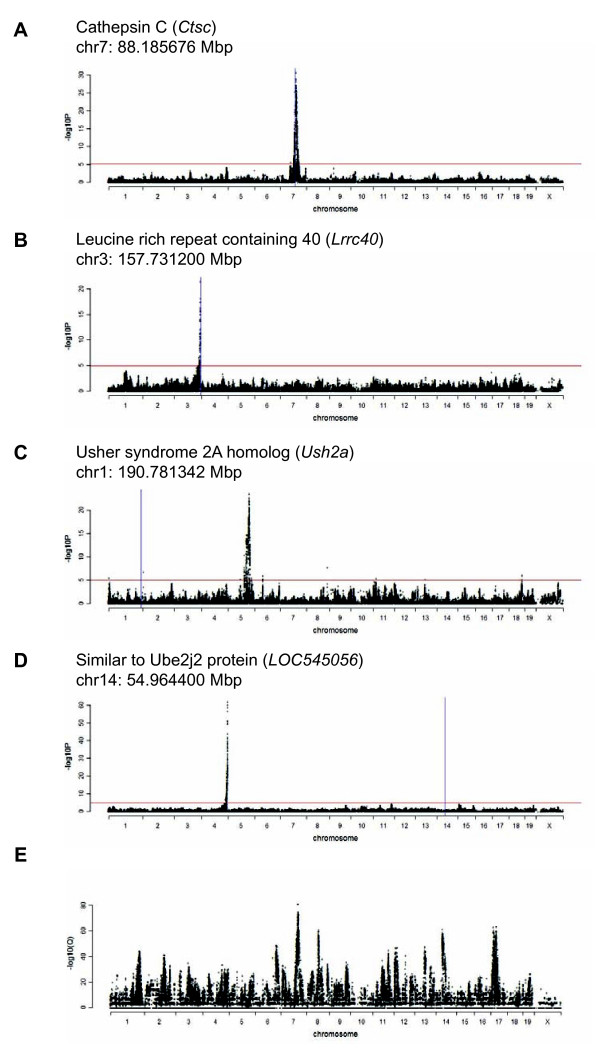
**Examples of *cis *and *trans *eQTLs in hippocampus and striatum**. **A) **Hippocampus *cis *eQTL. **B) **Striatum *cis *eQTL. **C) **Hippocampus *trans *eQTL. **D) **Striatum *trans *eQTL. Red horizontal line represents genome wide significance threshold of FDR < 5% for each tissue. Blue vertical line represents gene position. **E) **Degree of overlap between tissues for probes regulated by each marker between tissues at FDR < 5%. Significance shown as - log_10_(*Q*).

In the hippocampus, we mapped 481,099 *trans *eSNPs regulating a total of 5,325 unique probes, while in the striatum, we mapped *trans *619,418 eSNPs regulating a total of 15,348 unique probes. Using a counting algorithm (**METHODS**), we estimated these numbers corresponded to a total of 19,876 *trans *eQTLs in the hippocampus and 60,150 *trans *eQTLs in the striatum. Genome-wide probe/marker plots for each significant eSNP are provided in the Supplementary materials (Additional file 1 Figures S4 and S5). Selected *cis *and *trans *eQTLs from each tissue are shown in Figure 3A - 3D.

Comparison of our data with a recent eQTL survey in the hippocampus using heterogeneous stock mice [25] showed significant preservation of *cis *eQTLs (χ^2 ^= 1,171, *df *= 1, *P *= 1.1 × 10^-256^), while *trans *eQTLs did not show significant overlap. This discrepancy could be due to weaker effect sizes for *trans *eQTLs in general compared to *cis *or due to differing thresholds for significance. Previous studies also found that *trans *eQTLs replicated less frequently than *cis *[26,27]. A recent study of liver using the HMDP [9] found 2,691 *cis *eQTLs and 3,174 probes with at least one *trans *eQTL with *P *< 4.1 × 10^-6^. We detected similar numbers of *cis *eQTLs but more *trans *loci, even though the same significance threshold was employed for both types of eQTL. This discrepancy suggests differences in the regulatory networks of hepatic versus neural tissue and may reflect greater transcriptional complexity in the brain.

To survey whether *trans *gene regulation in hippocampus was similar to that found in the striatum, we compared the probes regulated by each marker across the two tissues. Using a 2 × 2 contingency table, we determined if a probe was regulated by each marker in the hippocampus or not (surpassing a global FDR of 5%) and regulated by the same marker in the striatum or not. There was a significant overlap in the genes regulated by each marker across the tissues (Fisher's Exact Test, *df *= 1, median omnibus -log_10_(*Q*) = 4.1), suggesting strong similarities in the regulatory networks of the two tissues. A genome-wide plot of the -log_10_(*Q*) of the degree of overlap between tissues for genes regulated by each marker between tissues is shown in Figure 3E. Some markers clearly show better preservation of regulated probes than others. For instance a SNP on chromosome 7 at 104.063430 Mb regulates 33 unique genes in the hippocampus and 36 genes in the striatum, with 29 of the genes in common. These hubs may have strong control of expression across different tissues. Despite the significant overlap, differences in regulation are likely important in delineating the cellular disparity between hippocampus and striatum.

### Weighted gene correlation network analysis (WGCNA)

We looked at the large scale organization of gene co-expression networks in the hippocampus and striatum microarray datasets. Weighted gene co-expression network analysis is a data reduction method that groups genes into modules in an unsupervised manner based on self-organizing properties of complex systems. These co-expression networks are based on topological overlap between genes while considering the correlation genes have with each other and the degree of shared connections within the network. This method has been used in several recent systems genetics studies to reveal functional gene networks [28,29].

We identified 30 modules in hippocampus containing 39 to 8,445 genes and 25 modules in the striatum containing 34 to 14,582 genes (Additional file 1 Table S4). The largest module in each tissue is the grey module which is reserved for genes that do not separate into any other modules (noise genes). The hippocampus expression data organized into five more modules than the striatum. This finding could reflect a greater cellular heterogeneity of the hippocampus compared to the striatum, as module construction can tease apart patterns of differential expression in mixtures of cell types [30]. There were other differences in co-expression networks between the two tissues. For instance the sienna3 module in the hippocampus was not preserved in striatum. This module was significantly enriched in neuropeptide hormone activity (*Q *= 6.25 × 10^-6^) and oxygen binding (*Q *= 3.68 × 10^-4^) indicating that these molecular classes may play important roles in hippocampal function.

**Figure 4:**
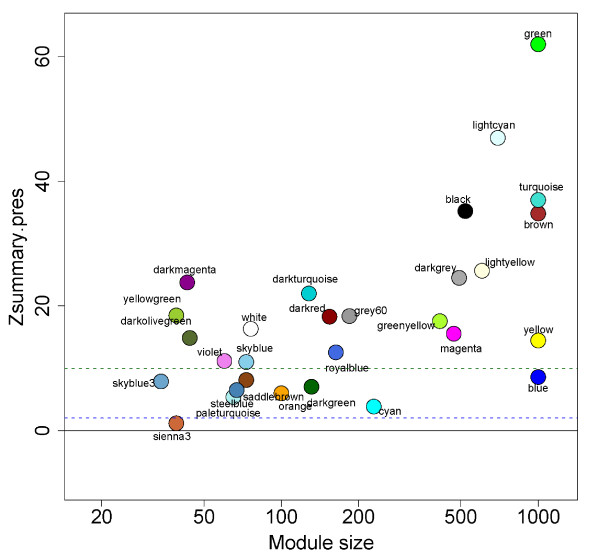
**Gene co-expression module preservation across hippocampus and striatum**. Modules were constructed separately for each tissue and preservation assessed by Zsummary score using hippocampus modules as reference set. Larger modules tended to be better preserved across tissues.

To evaluate the degree of module conservation across the hippocampus and striatum, we calculated Z scores for preservation of each module using the hippocampus as a reference. The Zsummary statistic encapsulates evidence that a network module is preserved between a reference and a test network based on aspects of within-module network density and connectivity patterns [31]. Lower Z.summary.pres scores imply module differences while larger ones indicate preservation. Figure 4 demonstrates that most gene co-expression modules showed some degree of preservation across hippocampus and striatum, with larger modules showing better preservation than smaller ones.

**Figure 5:**
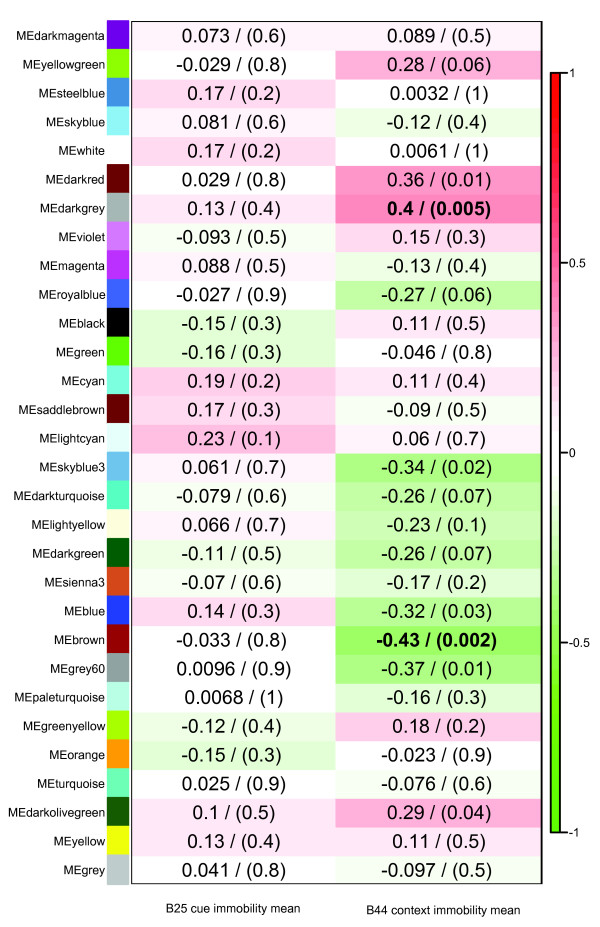
**Correlation of module eigengenes with cued and context immobility phenotypes in the hippocampus**. Columns represent cued and context immobility phenotypes and the rows represent MEs. Correlations between MEs and phenotype represented by colors ranging from red (high positive correlation) to green (high negative correlation). Correlation coefficient shown for each comparison with corresponding *P *value in parentheses. Two highlighted modules shown in boldface.

The gene expression properties of each of these modules can be condensed into module eigengenes (MEs) which represent the first principal component of each module [32,33]. By correlating these MEs to behavioral phenotypes, we were able to identify groups of genes with relationships with aspects of conditional fear. Figure 5 shows the correlation of each ME in the hippocampus with the behavioral phenotypes of cued and context immobility (B25 and B44). We focused on hippocampus, as this tissue has been previously implicated in learning, memory, and fear [34].

The context immobility phenotype (B44) showed the strongest correlations with two MEs in the hippocampus: brown (r = -0.43, *P *= 0.002, *Q *= 0.07) and darkgrey (r = 0.4, *P *= 0.005, *Q *= 0.08). We focus on these two modules for further analysis and annotate them context fear module 1 (CF1) and context fear module 2 (CF2) respectively. Notably, no MEs showed significant correlations with cued immobility (B25) even though cue and context immobility phenotypes clustered together (Additional file 1 Figure S1). This observation is consistent with the biology of cued immobility which relies on the amygdala but is hippocampal dependent [35].

We looked for functional enrichment of specific gene ontologies (GO) in the two selected context fear modules using the program GOEAST, which provides an FDR corrected *Q *value [36] score for enrichment in each category. The most highly represented ontologies are shown in Additional file 1 Tables S5 and S6. Genes in the intracellular portion of the cell were enriched in both modules (CF1: *Q *= 1.54 × 10^-16^, CF2: *Q *= 2.33 × 10^-8^), as were those involved in the mitochondrion (CF1: *Q *= 4.38 × 10^-6^, *Q *= 2.1 × 10^-3^). By contrast, classes of genes involved in metabolic processes and gene expression were specific to CF1. Genes involved in protein targeting and the rough endoplasmic reticulum were prominent in CF2 but not in CF1. Results of correlations between MEs and all quantified behavioral traits for the hippocampus and striatum are provided in Additional file 1 (Figures S6 and S7).

Genes within each module are prioritized according to their intramodular connectivity (the sum of connection strengths with other genes within the network). Those with a high degree of connectivity are considered hubs and can be viewed as important players in molecular pathways. There was a high correlation between the intramodular connectivity measures of each gene across the hippocampus and striatum (*r *= 0.53, *P *< 2.2 × 10^-16^) indicating strong similarities in the transcriptional networks of these neural tissues.

The gene mitogen-activated protein kinase 1 (*Map2k1*) was one of the most highly connected genes in CF1 and has been previously implicated in long-term synaptic plasticity and memory [37]. The gene proteasome (prosome, macropain) 26 S subunit, non-ATPase, 6 (*Psmd6*) acted as another hub in CF1, while in CF2, the genes ubiquitin-conjugating enzyme E2A (*Ube2a*), nuclear factor I/B (*Nfib*), and ubiquitin specific peptidase 33 (*Usp33) *had the strongest intramodular connectivity and served as hubs for this module. These results suggest a role for targeted protein degradation in pathways associated with context dependent fear, consistent with a recent study that showed that synaptic protein degradation through polyubiquitination underlies the destabilization of retrieved fear memory [38]. Other co-expressed genes identified in these modules may also play critical roles in the molecular mechanisms governing learning and memory. Complete details for the gene co-expression network analysis for each tissue and the corresponding measures of intramodular connectivity for each gene can be found in Supplementary materials (Additional file 2).

### MEs as quantitative traits

Each module eigengene can be considered a quantitative trait, allowing for mapping of SNPs associated with variation in groups of co-expressed genes. This strategy reveals loci that perturb the expression of gene modules with hopes of uncovering key drivers for traits of physiological relevance [39]. Mapping results that survive a Bonferroni correction for all 101,629 markers are summarized in Table 2. Loci regulating six MEs in the hippocampus were mapped, of which four were preserved in the striatum and two were specific to hippocampus. The first hippocampal specific locus regulated the darkolivegreen module and mapped to a SNP on chromosome 7 within the intron for the gene TEA domain family member 1 (*Tead1*), a gene known to be associated with transcription factor complexes. This module was enriched in the cellular component flotillin complex (*Q *= 4.90 × 10^-6^) and the molecular function calmodulin-dependent protein kinase activity (*Q *= 4.77 × 10^-5^). The second hippocampal specific locus regulated the white module and mapped to a SNP on chromosome 1 at 173.121821 Mb. This module consisted of genes involved in the positive regulation of the acute inflammatory response to antigenic stimulus (*Q *= 4.54 × 10^-5^).

**Table 2:** Loci regulating module eigengenes and significance

Hippocampus module	Striatum module	Chromosome	Base Position	Hippocampus *P *valve	Striatum *P *valve
darkmagenta	paleturquoise	17	24,843,527	9.38 × 10^-28^	1.75 × 10^-22^
yellowgreen	saddlebrown	17	33,901,252	2.31 × 10^-26^	3.34 × 10^-32^
skyblue3	skyblue	8	125,688,170	1.93 × 10^-18^	3.52 × 10^-15^
Orange	steelblue	14	50,200,200	1.87 × 10^-31^	9.56 × 10^-42^
darkolivegreen	-	7	108,611,544	2.28 × 10^-29^	-
White	-	1	173,121,821	1.18 × 10^-21^	-

The module with the strongest association to physiologically relevant GO categories that also possessed regulatory loci for both tissues was the yellowgreen module in the hippocampus (saddlebrown in striatum). This module was enriched in antigen processing and presentation (*Q *= 1.61 x10^-21^) and MHC protein complex (*Q *= 3.10 × 10^-19^). This module may play a role in synaptic remodeling, as neuronal MHC class I molecules were recently found to regulate synapses in the central nervous system in response to activity [40]. Interestingly, the regulatory locus for this module was identical for hippocampus and striatum. A potential candidate for this locus was flotillin 1 (*Flot1*), a gene with a *cis *eQTL in both hippocampus and striatum ~24 kb away from this peak marker. This gene product has been found to accumulate in tangle-bearing neurons of Alzheimer's disease [41] and may play a role in learning. In addition, the flotillin complex featured in the darkolivegreen module regulated by a hippocampal locus (above). Other genes in these identified modules should be examined as potential players in the molecular pathways for fear conditioning.

### Network edge orienting: prioritizing directed trait networks

**Figure 6:**
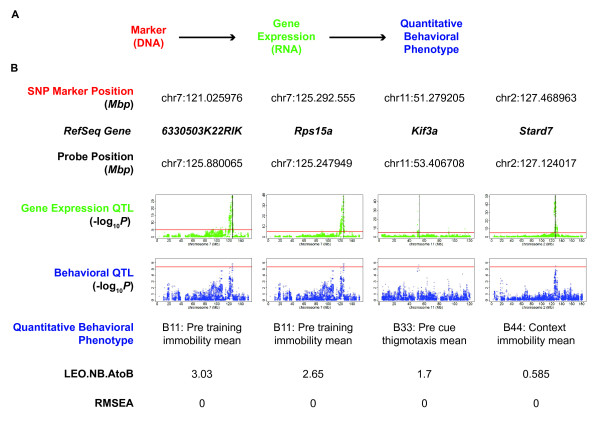
**Selected causative genes in the hippocampus found using network edge orienting**. **A) **Model fitted by NEO software implicates a marker (DNA) as causal for a phenotypic trait through expression of a gene (RNA) **B) **Thresholds for FDR < 5% shown as red horizontal lines. Vertical black lines indicate the start position of the gene. *6330503K22RIK, Rps15a*, *Kif3a*, and *Stard7 *are genes with local markers that perturb gene expression levels, which in turn contribute to fear phenotypes.

To look for relationships between genetic variation, differences in gene expression, and behavioral phenotypes, we employed the Network Edge Orienting (NEO) [42] algorithm. Using SNP markers as causal anchors, NEO assigns directionality to trait networks and provides a way to prioritize genes with expression profiles that are coincident with quantitative behavioral phenotypes (Figure 6A).

We performed a NEO single marker analysis on markers with an FDR < 10% in the behavioral QTL mapping. The software uses structural equation modeling to fit five models: causal, reactive, independent, and two confounded models. NEO compares the best fitting model relative to the next best fitting model, yielding a log_10 _likelihood ratio, LEO.NB.AtoB, for each significant SNP for each of the behavioral endpoints. Values greater than 0.3 for this score indicate that the causal model fits the input data twice as well as the next best model; a score of 1 indicates a ten-fold better fit. The measure RMSEA.AtoB is an index of model fit, with values < 0.05 representing a good fit.

Figure 6B shows the results of NEO analysis in the hippocampus. The results indicate that two SNP markers located on chromosome 7 regulate the expression of two nearby genes on chromosome 7 (*630503K22RIK *and *Rps15a*) which in turn influence the immobility of the animals before training (B11: Pre training immobility mean).

Genetic variation at a SNP on chromosome 11 at 51.279205 was also shown to influence the expression of the nearby gene kinesin-like protein 3A (*Kif3a*) which then contributed to variation in thigmotaxis (B33: Pre cue thigmotaxis mean). *Kif3a *is a kinesin gene involved in moving axon cargo [43] and has been implicated in amyotrophic lateral sclerosis, a disease involving degeneration of motor neurons [44].

Variation at a SNP on chromosome 2 resulted in a change in expression of the gene START domain-containing 7 (*Stard7*) which then influenced immobility induced by a novel context (B44 Context immobility). The genes *6330503K22RIK *and *Kif3a *also appear as strong candidates for fear related behavior in the NEO analysis for the striatum (Additional file 1 Figure S8), underscoring the similarity of transcriptional regulation in the two tissues.

## Discussion

Fear conditioning provides an opportunity to survey a range of clinically relevant processes including short and long-term memory, context generalization, and memory extinction, making it an efficient tool with which to probe the genetics of fear dependent behavior. To map fear related QTLs, we subjected a population of inbred mouse strains to a standard fear conditioning procedure and follow-up memory tests. We then combined behavioral phenotype data with SNP genotypes and tissue specific gene expression to search for candidate genes and related networks associated with fear phenotypes. Across 48 behavioral endpoints, we mapped a total of 27 QTLs, highlighting the complexity of behavioral regulation and showcasing the value of HMDP for mapping fear loci.

The inbred strains of the HMDP were not randomly selected, but were, in fact, carefully chosen to avoid, insofar as possible, high correlation of non-linked genome segments. Nevertheless, there are some shared segments across the genome due to bottlenecks in the breeding and the history of the strains. EMMA endeavors to correct for these artifacts in the association analysis. However, some caution should be applied to the interpretation of the mapping results, since bias may remain which cannot be overcome by the analysis of the data.

The strongest behavioral QTL in our investigation was for the phenotype cue immobility and had two peak markers on chromosome 7. These markers were located in the adjacent genes *Tyr *and *Grm5 *and had identical *P *values of 4.4 × 10^-9^, yet there were recombination breakpoints between them. Many HMDP strains have mutations in *Tyr *and are albino, resulting in possibly learning and memory deficits due to decreased visual acuity. However, a study that examined this allele specifically showed that it plays only a minor role in cue immobility and that additional loci are likely to influence fear conditioning [16]. *Grm5 *is an attractive candidate gene for this locus, since it has previously been shown to be involved in hippocampal LTP.

We surveyed the architecture of transcriptional regulation across two brain regions. We found a smaller number of *cis *and *trans *eQTLs in the hippocampus than in the striatum. This diminution may be caused by signal dilution due to the heterogeneous cellular nature of the hippocampus. However we found that the *cis *and *trans *eQTLs in the two tissues overlapped significantly, indicating that DNA polymorphism has a robust effect in modulating gene expression across tissues.

By simplifying the gene expression data into modules, we identified groups of genes that are related to fear related behavior. Two such modules in the hippocampus (CF1 and CF2) showed strong correlations with context-dependent fear measures, allowing identification of networks of genes whose co-expression co-varied with fear phenotypes across the HMDP. We assigned priorities to genes within each module based on their level of intramodular connectivity and mapped loci responsible for regulating MEs in both hippocampus and striatum. Cued and context immobility were phenotypically similar as they clustered together in the behavioral dendrogram. However, the two identified modules did not show strong correlations with cued fear, confirming suggesting that the two different types of fear are expressed through different neural and/or molecular pathways.

A hub gene in CF1 (Psmd6) and two of the most highly connected genes in CF2 (*Ube2a *and *Usp33*) have been shown to play roles in ubiquitination. Interestingly, others have shown that ubiquitin-mediated proteolysis is involved in initiating long-term stable memory, as both specific removal of specific inhibitory proteins and gene induction are likely to be critical players in fear conditioning [45]. Other components in these modules may be implicated by association in these genetic pathways and provide attractive targets for further investigation.

Structural equation modeling allowed us to identify single markers that influenced the expression of single genes which in turn influence fear related phenotypes. We identified five genes with causal relationships for fear-related phenotypes in the hippocampus and striatum including *6330503K22RIK, Rps15a, Kif3a, Stard7*, and *Plvap*.

## Conclusion

In summary, looking at expression patterns in genes and groups of genes in various neural tissues has helped to elucidate the complex molecular networks contributing to fear dependent behavior. While the current approach yielded several potential loci and candidate genes, additional inbred strains would provide increased power for more comprehensive mapping. Next generation sequencing technologies and proteomics should afford even deeper views of genetic polymorphism and expression as we continue to refine gene networks of fear neurobiology.

## Methods

### Mouse population

Male mice from the Mouse Diversity Panel (HMDP) were used for all behavioral analyses. This panel of mice consists of 100 inbred strains comprised of 29 classical inbred strains paired with three sets of RI strains selected for diversity [9]. All mice (n = 700) were obtained through Jackson Laboratory at approximately 55 days old then housed for a 14-day acclimation period prior to testing. Mice were housed in groups (3-4 per cage) under a 12hr/12hr day/night cycle with ad lib access to food and water. All behavioral testing was conducted during the day portion of the cycle, between the hours of 10 AM and 4 PM. Protocols conformed to NIH Care and Use Guidelines and were approved through the UCLA Animal Research Committee. Mice were housed in their covered home cages and placed in an adjacent holding room. Auditory background stimulus in the form of white noise (80db) was delivered through overhead speakers. Previous unpublished observation showed no evidence of orienting response, or any behavioral responses to stimulus presentation while in the holding room [15].

### Fear Conditioning

All HMDP strains were exposed to a fear conditioning procedure followed by two independent memory tests. Parameters and procedures were identical to those previously described [15]. On each test day, mice were wheeled to a holding room for a 30 min acclimation period prior to testing. Each mouse was tested individually and then transferred to a holding cage. On day 1, mice were placed in a 25 cm × 20 cm conditioning chamber with grid floors and white plexiglass. Following a 3 minute exploration period, mice received three auditory conditional stimuli (CS; 2000Hz, 15 seconds, 80 dB) co-terminating with footshock unconditional stimulus (US; 0.75 mA, 1 second), delivered with an inter-trial interval (ITI) of 1 minute. Mice were removed 2 minutes following the final US. On day 2, contextual fear was assessed. Mice were then returned to the conditioning chamber under conditions identical to day 1. Neither the CS nor US was presented during an 8 minute test. On day 3, cued fear was assessed following a contextual shift. Mice were placed in a novel, rectangular activity chamber (50 cm × 25 cm), given a 3 minute exploration period followed by a series of ten CS presentations (ITI 1 min), then removed from the chamber 1 minute following the final CS. No US were presented during this test. This apparatus was cleaned with 70% ethanol between tests.

### Behavioral Data Analysis

Behavior was recorded digitally from a camera mounted above each test chamber, then digitized at 15 frames per second with the EthoVision Pro tracking system (Noldus Information Technology). For each mouse a total of 48 unique endpoints were quantified automatically with EthoVision software (Additional file 1 Table S1). Varying numbers of biological replicates were obtained for each strain (ranging from *n *= 3 to *n *= 16, mean = 7.3). These measures were designed to characterize multiple dimensions of defensive behavior. The methodology and rationale behind these measures has been discussed previously [15].

Mean performance for each endpoint was determined by either collapsing across the entire test session for context fear endpoints or across specific test phases for fear conditioning (pre-US, post-US) and cued fear test (pre-CS, CS) endpoints. The pre-US period consisted of the 3 minutes prior to the initial CS presentation, while the post-US period encompassed the 4.25 minute interval between the first US presentation and removal from the chamber. Likewise, the pre-CS period spanned the 3 minutes prior to CS presentation, and the CS period covered the 12.5 minute period between the first CS presentation and removal from the chamber. Measures reflecting rate changes were quantified by analyzing time course data within individual test phases.

For the context test, endpoint rate changes were calculated as the percent change from the initial 2 minute epoch to the final 2 minute epoch. For multi-phase tests (training, cued fear test), rate changes were calculated as suppression ratios based on mean values from the relevant test phases (pre/(pre+post)). Strain means were calculated and served as the behavioral phenotypes for downstream analysis. Velocity is the mean rate of movement in any given interval (e.g. cm/s), while mobility is the time spent mobile, expressed as a percentage of total time.

### Genotype analysis

The classical inbred and RI strains were genotyped previously [9] by the Broad Institute (classical) and the Wellcome Trust Center for Human Genetics (RI). The genotypes of the RI lines at the Broad SNPs were imputed from the Wellcome Trust genotypes. Only SNPs with a minor allele frequency greater than or equal to 10% were used in the analysis to minimize false positives due to small sample size. All genome coordinates are based on NCBI build 35 (mm7) of the mouse genome.

### Behavioral QTL mapping

Using the collected behavioral phenotypes, we performed a genome-wide association test using the software package EMMA (Efficient Mixed-Model Association) [12]. This program calculates *P *values which quantify the degree of association between each probe-marker pair while correcting for confounding effects of population structure and genetic relatedness between strains in the panel. We used a genome-wide *Q *value threshold of 5% [23] which corresponds to a *P *value of 4.1 × 10^-6 ^. To count the number of significant QTL, the genome was divided into bins of 2Mb. If significant markers were found in adjacent bins, markers were combined and counted as a single QTL.

### Tissue harvesting

Brains were removed from each animal after euthanasia. Hippocampus and striatum were dissected out and flash frozen in liquid nitrogen. RNA was extracted from each sample using the Qiagen RNeasy kit.

### Microarray data collection

Gene expression levels were quantified using Illumina Mouse-Ref 8 v2.0 Expression BeadChip microarrays. The data were normalized using the rank invariant option in the software package BeadStudio (Illumina) [46]. The microarray data are available at the Gene Expression Omnibus (GEO) http://www.ncbi.nlm.nih.gov/geo/ under accession number GSE26500.

### Expression quantitative trait loci (eQTL) mapping

Using the marker genotype information from the HMDP and RNA expression data from hippocampus and striatum, we performed a genome-wide association test for each of the 25,697 probes (genes) on the microarray compared to each of the 101,629 SNP markers using the software package EMMA. Markers within 2 Mb of the probe position for each gene were considered *cis *(local), while those greater than 2 Mb from the probe position were considered *trans *(distant). Genome-wide significance thresholds were determined by calculating the *P *value corresponding to a Benjamini and Hochberg corrected FDR of 5% [23]. To count the number of significant *trans *loci, we divided the genome into bins of 2 Mb in width and counted whether or not a marker that surpassed an FDR of 5% was observed in the bin or not. If adjacent bins contained at least one significant marker, the bins were combined together and counted as a single locus.

### Gene ontology enrichment analysis

Groups of identified genes were checked for enrichment in gene ontology categories using the package GOEAST [24]. Significance was reported as *Q *values (*P *value corrected false discovery rates [36]).

### Identification of gene co-expression modules associated with behavioral phenotypes

We used the R package WGCNA [47] to create gene co-expression modules. The input data consisted of gene expression data from the hippocampus (n = 94) and the striatum (n = 94). This program created modules or clusters of highly correlated genes in each tissue separately. For each of the modules, the program produced a module eigengene (ME) which enabled us to find relationships of modules with behavioral phenotypes.

### Module preservation

We used the modulePreservation function from the WGCNA library to calculate module preservation statistics [31]. The Zsummary is derived from seven underlying statistics that measure preservation of various aspects of within-module network density and connectivity patterns. The underlying preservation statistics are based on permutation tests and their values represent evidence that a module is significantly better preserved between the reference and test networks than a randomly sampled group of genes of the same size. A Zsummary < 2 indicates no evidence of module preservation, 2 < Zsummary < 10 indicates weak to moderate module preservation, and Zsummary > 10 indicates strong preservation.

### Network edge orienting

Markers surpassing a FDR threshold of 10% in the behavioral QTL analysis along with gene expression data for hippocampus and striatum were used as input to the Network Edge Orienting (NEO) software package in R [42]. We selected marker, gene, and phenotype combinations that yielded a LEO, NB.AtoB score > 0.3 and RMSEA.AtoB score < 0.05 for further analysis.

## Authors' contributions

CCP participated in the analysis of the expression and behavior data and drafting of the manuscript. GDG participated in the analysis of the behavior data and drafting of the manuscript. SdJ, AG, PL, AL, EE and SH participated in the statistical analyses. BB, CRF and AK participated in sample collection and analysis. AJL, RAO, EE, and SH participated in the design of the study. DJS conceived of the study, and participated in its design and coordination. All authors read and approved the final manuscript.

## Supplementary Material

Additional file 1 **Supplementary Methods, Tables and Figures**. The Supplementary Methods describe further analyses of fear phenotypes in the HMDP and gene regulation hotspots from the eQTL mapping. Supplementary Tables are Table S1, Classification of quantified behavioral phenotypes; Table S2, Top 100 cis eQTLs in hippocampus; Table S3, Top 100 cis eQTLs in striatum; Table S4, Gene co-expression modules; Table S5, Functional classification for genes in context fear module 1; Table S6, Functional classification for genes in context fear module 2. Supplementary Figures are Figure S1, Cluster dendrogram by behavioral phenotype across HMDP; Figure S2, Mapped locus for cue immobility on chromosome 7; Figure S3, QTL plots for 48 tested behavioral phenotypes after EMMA correction for population structure; Figure S4, Hippocampus eQTLs; Figure S5, Striatum eQTLs; Figure S6, Hippocampus module-trait correlations; Figure S7, Striatum module-trait correlations; Figure S8, Striatum NEO results. Click here for file

Additional file 2 **Gene connectivity and module information**. Table provides details of gene co-expression network analyses for each tissue and corresponding measures of intramodular connectivity for each gene. Click here for file
