# Supplementary material for: Gene networks associated with conditional fear in mice identified using a systems genetics approach
Source: BMC Syst Biol. 2011 Mar 16;5:43. doi: 10.1186/1752-0509-5-43 (PMC3070648; doi:10.1186/1752-0509-5-43)
Supplement: Additional file 1 — Supplementary Methods, Tables and Figures. The Supplementary Methods describe further analyses of fear phenotypes in the HMDP and gene regulation hotspots from the eQTL mapping. Supplementary Tables are Table S1, Classification of quantified behavioral phenotypes; Table S2, Top 100 cis eQTLs in hippocampus; Table S3, Top 100 cis eQTLs in striatum; Table S4, Gene co-expression modules; Table S5, Functional classification for genes in context fear module 1; Table S6, Functional classification for genes in context fear module 2. Supplementary Figures are Figure S1, Cluster dendrogram by behavioral phenotype across HMDP; Figure S2, Mapped locus for cue immobility on chromosome 7; Figure S3, QTL plots for 48 tested behavioral phenotypes after EMMA correction for population structure; Figure S4, Hippocampus eQTLs; Figure S5, Striatum eQTLs; Figure S6, Hippocampus module-trait correlations; Figure S7, Striatum module-trait correlations; Figure S8, Striatum NEO results. [file 1752-0509-5-43-S1.PDF]

Supplementary materials:

Gene networks associated with conditional fear in mice identified using a systems genetics approach

Christopher C. Park[1*], Greg D. Gale[1*], Simone de Jong[2], Anatole Ghazalpour[3], Brian Bennett[4], Charles R. Farber[3#], Peter Langfelder[4], Andy Lin[1], Arshad Khan[1], Eleazar Eskin[4,5], Steve Horvath[4], Aldons J. Lusis[3,4], Roel A. Ophoff[2,4,6], and Desmond J. Smith[1§]

[1]Department of Molecular and Medical Pharmacology, David Geffen School of Medicine, University of California, Los Angeles, CA 90095, USA
[2]Department of Medical Genetics and Rudolf Magnus Institute of Neuroscience, UMC Utrecht, 3584 CG, Utrecht, The Netherlands
[3]Department of Medicine - Cardiology, David Geffen School of Medicine, University of California, Los Angeles, CA 90095, USA
[4]Department of Human Genetics, David Geffen School of Medicine, University of California, Los Angeles, CA 90095, USA
[5]Department of Computer Science, University of California, Los Angeles, CA 90095, USA
[6]University of California, Los Angeles, Center for Neurobehavioral Genetics, David Geffen School of Medicine, CA 90095, USA

[§]Corresponding author
[*]These authors contributed equally to this work
[#]Current address: Center for Public Health Genomics, School of Medicine, University of Virginia, VA 22908, USA

Email addresses:
CCP: ccpark@ucla.edu
GDG: ggale_uc@gmail.com
SDJ: s.dejong-6@umcutrecht.nl
AG: aghazalp@ucla.edu
BB: BBennett@mednet.ucla.edu
CRF: crf2s@virginia.edu
PL: peter.langfelder@gmail.com
AL: sonic_22@yahoo.com
AK: akhan@mednet.ucla.edu
EE: eeskin@ucla.edu
SH: SHorvath@mednet.ucla.edu
AJL: JLusis@mednet.ucla.edu
RAO: ROphoff@mednet.ucla.edu
DJS: DSmith@mednet.ucla.edu

TABLE OF CONTENTS

SUPPLEMENTARY METHODS

1. Fear phenotypes in the HMDP

2. Hotspots of gene regulation found in eQTL mapping

SUPPLEMENTARY TABLES

Table S1. Classification of quantified behavioral phenotypes (48 total)

Table S2. Top 100 *cis* eQTLs, hippocampus

Table S3. Top 100 *cis* eQTLs, striatum

Table S4. Gene co-expression modules

Table S5. Functional classification for genes in CF1

Table S6. Functional classification for genes in CF2

SUPPLEMENTARY FIGURES

Figure S1. Cluster dendrogram by behavioral phenotype across HMDP.

Figure S2. Mapped locus for B25 cue immobility on chromosome 7.

Figure S3. QTL plots for 48 tested behavioral phenotypes after EMMA correction for population structure.

Figure S4. Hippocampus eQTLs.

Figure S5. Striatum eQTLs.

Figure S6. Hippocampus Module-trait correlations.

Figure S7. Striatum Module-trait correlations.

Figure S8. Striatum NEO results.

Fear phenotypes in the HMDP

Across the population, context and cued fear tests generated comparable levels of immobility (mean=34.2% and 30.7%, respectively). Immobility varied considerably between strains and test phases. High variability in fear between context and cued tests may indicate qualitative differences in how memory is structured, so we surveyed within-strain percent change in immobility across the two fear tests. The vast majority of strains (90%) exhibited between-test mean ratios of twice to one-half the immobility in one test vs. the other. The remaining strains showed strong bias for cued immobility, reflecting negligible levels of context fear normal cued fear. Collectively, the HMDP strains exhibited levels of conditioned fear consistent with previous reports and indicative of normal sensory and memory processes.

To assess potential interactions between immobility and other potentially confounding behavioral tendencies in HMDP strains, we examined the relationships between velocity, path shape and place preference during the 3 min pre-training period and immobility from subsequent test phases. Hyperactivity may directly compete with immobility leading to an underestimation of conditional fear. Similarly, highly variable path shape or overly defensive place preference strategies may suggest elevated anxiety. Pre-training velocity was negatively correlated with immobility in both the contextual (r= -0.10, $P < 0.005$) and cued fear tests ($r = -0.15$, $P < 0.0001$). Path shape was not correlated with context immobility (r= 0.001, $P > 0.97$) but was correlated with cued immobility ($r = 0.14$, $P < 0.0001$), while thigmotaxis, a common index of anxiety, was correlated with context ($r = 0.16$, $P < 0.0001$), but not cued immobility ($r = 0.04$, $P > 0.33$). The pattern of correlations is consistent with a recent report demonstrating that anxiety can be predictive of conditioned fear levels [1].

Despite the observed correlations, these measures explain a relatively low proportion of total immobility variance and, as such, strain differences in immobility likely reflect significant strain-specific variability in fear acquisition and/or expression processes.

Hotspots of gene regulation found in eQTL mapping

To look for hotspots (hubs) of gene regulation, we counted the number of genes regulated by each marker for each tissue. Assuming that the distribution of genes regulated by each marker follows a Poisson distribution ($\lambda = 4.20$, hippocampus; $\lambda = 5.35$, striatum), 10,286 markers were considered hubs in hippocampus while 9,536 markers were hubs in the striatum. The primary hotspot in the hippocampus was a SNP on the X chromosome at 138.602949 Mb. This marker regulated 102 unique probes ($Q = 0$) and is ~5.5 kb upstream of the gene *Dcx* (doublecortin, a neuronal migration protein). The second strongest hotspot was located on chromosome 7 at 81.834838 Mb, ~36 kb from the gene *Vmn2r73* (vomeronasal 2, receptor 73) and regulated 67 genes ($Q = 0$). The third most prominent hotspot in the hippocampus was located on chromosome 18 at 67.788308 within an intron of the gene *Tubb6* (tubulin, beta 6), a gene with a *cis* eQTL. This hotspot regulated 64 genes ($Q = 0$).

Interestingly the hotspots in the striatum tended to regulate more genes. The primary hotspot in the striatum regulated 973 ($Q = 0$) genes and was located on chromosome 16 at 74.454130 Mb, ~46 kb upstream of the gene *Robo2* (roundabout homologue 2, involved in axon guidance). The secondary hotspot regulated 943 ($Q = 0$) genes and was located on chromosome 5 at 97.044090 Mb ~54 kb from the *Antxr2* (anthrax toxin receptor 2). The

third strongest hotspot regulated 611 (Q = 0) genes and was located on chromosome 4 at 78.390172 Mb, ~ 1.8 Mb upstream of the gene *Tyrp1* (tyrosinase related protein 1).

REFERENCES

1. Lopez-Aumatell R, Vicens-Costa E, Guitart-Masip M, Martinez-Membrives E, Valdar W, Johannesson M, Canete T, Blazquez G, Driscoll P, Flint J *et al*: **Unlearned anxiety predicts learned fear: a comparison among heterogeneous rats and the Roman rat strains**. *Behav Brain Res* 2009, **202**(1):92-101.

Table S1. Classification of quantified behavioral phenotypes (48 total)

Phenotype name	Day	Time	Class	Behavior	Type	Additional Info
B1 pre training thigmotaxis mean	1	pre	training	thigmotaxis	mean	
B2 post training thigmotaxis mean	1	post	training	thigmotaxis	mean	
B3 pre training thigmotaxis mean distance to center	1	pre	training	thigmotaxis	mean	distance to center
B4 post training thigmotaxis mean distance to center	1	post	training	thigmotaxis	mean	distance to center
B5 pre training velocity mean	1	pre	training	velocity	mean	
B6 post training velocity mean	1	post	training	velocity	mean	
B7 training velocity habituation	1		training	velocity	habituation	
B8 pre training meander mean	1	pre	training	meander	mean	
B9 post training meander mean	1	post	training	meander	mean	
B10 training meander habituation	1		training	meander	habituation	
B11 pre training immobility mean	1	pre	training	immobility	mean	
B12 post training immobility mean	1	post	training	immobility	mean	
B13 pre training mobility mean	1	pre	training	mobility	mean	
B14 post training mobility mean	1	post	training	mobility	mean	
B15 pre cue thigmotaxis mean	3	pre	cue	thigmotaxis	mean	
B16 cue thigmotaxis mean	3		cue	thigmotaxis	mean	
B17 cue thigmotaxis extinction	3		cue	thigmotaxis	extinction	
B18 pre cue velocity mean	3	precue	cue	velocity	mean	
B19 cue velocity mean	3	cue	cue	velocity	mean	
B20 cue velocity habituation	3	cue	cue	velocity	habituation	
B21 pre cue meander mean	3	precue	cue	meander	mean	
B22 cue meander mean	3	cue	cue	meander	mean	
B23 cue meander habituation	3	cue	cue	meander	habituation	
B24 pre cue immobility mean	3	precue	cue	immobility	mean	
B25 cue immobility mean	3	cue	cue	immobility	mean	
B26 cue immobility extinction	3	cue	cue	immobility	extinction	
B27 cue mobility mean	3	precue	cue	mobility	mean	
B28 cue mobility mean	3	cue	cue	mobility	mean	
B29 cue mobility habituation	3	cue	cue	mobility	habituation	
B30 cue thigmotaxis mean distance to center	3	precue	cue	thigmotaxis	mean	distance to center
B31 cue thigmotaxis mean distance to center	3	cue	cue	thigmotaxis	mean	distance to center
B32 cue thigmotaxis habituation distance to center	3	cue	cue	thigmotaxis	habituation	distance to center
B33 pre cue thigmotaxis mean	3	precue	cue	thigmotaxis	mean	
B34 cue thigmotaxis mean	3	cue	cue	thigmotaxis	mean	
B35 cue thigmotaxis extinction	3	cue	cue	thigmotaxis	extinction	
B36 context thigmotaxis mean	2		context	thigmotaxis	mean	
B37 context thigmotaxis habituation	2		context	thigmotaxis	habituation	
B38 context thigmotaxis mean distance to center	2		context	thigmotaxis	mean	distance to center
B39 context thigmotaxis habituation distance to center	2		context	thigmotaxis	habituation	distance to center
B40 context velocity mean	2		context	velocity	mean	
B41 context velocity extinction	2		context	velocity	extinction	
B42 context meander mean	2		context	meander	mean	
B43 context meander extinction	2		context	meander	extinction	
B44 context immobility mean	2		context	immobility	mean	
B45 context immobility extinction	2		context	immobility	extinction	
B46 context mobility mean	2		context	mobility	mean	
B47 context mobility extinction	2		context	mobility	extinction	
B48 pain sensitivity	1		pain	sensitivity		

Table S2. Top 100 *cis* eQTLs, hippocampus

markerCount	minP	ILMN_Gene	RefSeq_ID	Probe_Id	chromosome	chrStart
85	9.637E-81	LRRC57	NM_025657.2	ILMN_2898944	2	120430648
273	5.506E-80	PSMB6	NM_008946.2	ILMN_2895862	11	70343427
59	7.359E-70	COPS8	NM_133805.3	ILMN_1218868	1	92509380
268	9.449E-69	PCOLN3	NM_145606.1	ILMN_2847502	8	126090554
122	4.047E-68	CASP9	NM_015733.4	ILMN_1217061	4	141371743
64	7.002E-68	RUSC2	NM_199057.2	ILMN_1214953	4	43439818
62	9.162E-67	RUSC2	NM_199057.2	ILMN_3159149	4	43439821
140	4.628E-65	IL17D	NM_145837.1	ILMN_2792502	14	58161838
35	1.826E-62	PDRG1	NM_178939.2	ILMN_1249417	2	152834935
365	1.734E-61	ZFP330	NM_145600.1	ILMN_2825109	8	85287669
183	2.98E-60	MRPL10	NM_026154.1	ILMN_2822622	11	96910086
107	1.241E-59	MPV17L	NM_033564.1	ILMN_2794258	16	13862714
324	1.367E-58	SSBP4	NM_133772.1	ILMN_2900827	8	73121502
257	6.545E-58	RBM13	NM_026453.1	ILMN_3009910	8	32625759
274	1.194E-56	PRDX2	NM_011563.2	ILMN_2638354	8	87864469
118	1.069E-54	SERPINA3H	NM_001034870.2	ILMN_2889832	12	105492461
177	7.378E-53	ME2	NM_145494.2	ILMN_1230263	18	73929996
146	4.8E-52	GPN2	NM_133884.1	ILMN_1214375	4	133147483
274	5.475E-52	PRDX2	NM_011563.2	ILMN_1242829	8	87864541
116	6.718E-52	PTCD3	NM_027275.3	ILMN_1228040	6	71831116
309	2.382E-51	SLC5A5	NM_053248.1	ILMN_1250582	8	73407570
296	1.796E-50	STARD7	NM_139308.1	ILMN_2676066	2	127124017
258	4.156E-49	NUDT6	NM_153561.2	ILMN_1253773	3	37310483
203	5.256E-49	TTC27	NM_152817.3	ILMN_1238733	17	75262828
212	8.843E-49	THUMPD1	NM_145585.1	ILMN_2722488	7	126858959
87	1.873E-48	TESK1	NM_011571.2	ILMN_1231930	4	43460830
67	2.386E-48	SEMA5A	NM_009154.1	ILMN_2604224	15	32625929
67	3.703E-48	SEMA5A	NM_009154.2	ILMN_2604226	15	32625932
319	4.289E-48	FCER1G	NM_010185.2	ILMN_2748875	1	173159942
176	6.1E-47	MBOAT2	NM_001083341.1	ILMN_1229343	12	25644557
247	1.221E-46	RSF1	NM_001081267.1	ILMN_2921163	7	104810161
64	1.238E-46	PAIP1	NM_145457.3	ILMN_2752569	13	120236564
115	1.615E-46	1700123O20RIK	NM_021437.1	ILMN_2597332	14	55309002
336	2.182E-46	UAP1	NM_133806.4	ILMN_2502542	1	172072552
20	2.474E-46	KIF3A	NM_008443.3	ILMN_2621148	11	53406708
134	4.696E-45	MRPS27	NM_173757.3	ILMN_2588199	13	100185149
100	8.993E-45	B3GALT6	NM_080445.4	ILMN_2687140	4	155364722
196	1.37E-44	ARV1	NM_026855.1	ILMN_2836173	8	127619994
21	1.612E-44	IGSF11	NM_170599.2	ILMN_2965399	16	39027008
78	2.411E-44	CCNG2	NM_007635.3	ILMN_2700233	5	93704893
112	3.227E-44	ENPP5	NM_032003.1	ILMN_1217118	17	44223312
158	4.925E-44	TASP1	NM_175225.3	ILMN_2714138	2	139659580
18	4.933E-44	GDPD3	NM_024228.2	ILMN_2893879	7	133914694
161	7.663E-44	PICALM		ILMN_2769656	7	97357127
256	2.612E-43	LOC100048020	XM_001479635.1	ILMN_2653881	4	137998107
21	1.369E-42	ZFP235	NM_019941.2	ILMN_2466926	7	24927893
44	2.24E-42	NLN	NM_029447.1	ILMN_2786567	13	104813546
181	2.418E-42	ZNHIT3	NM_001005223.1	ILMN_2914957	11	84727546
217	3.914E-42	BC026585	XM_129546.2	ILMN_1221257	1	159418777

9	6.955E-42	MRPS10	NM_183086.1	ILMN_2841840	17	47515340
176	9.693E-42	SC4MOL	NM_025436.1	ILMN_2823778	8	67610701
239	1.048E-41	EG381438	NM_198657.1	ILMN_2998738	3	37906223
360	1.432E-41	SAMD4	NM_001037221.1	ILMN_3128535	14	47721611
99	1.604E-41	SERPINA3N	NM_009252.2	ILMN_1246800	12	105652409
131	1.748E-41	CSRP1	NM_007791.4	ILMN_1260378	1	137648559
9	1.836E-41	ATP6V1D	NM_023721.2	ILMN_2647048	12	79944135
72	8.189E-41	CAP1	NM_007598.2	ILMN_2648661	4	122536456
234	2.198E-40	NUP133	NM_172288.1	ILMN_2981689	8	126428461
336	3.404E-40	RPL29	NM_009082.2	ILMN_2730005	9	106331900
244	3.821E-40	GPS2	NM_019726.3	ILMN_2730784	11	69728828
99	3.971E-40	AGPAT5	NM_026792.3	ILMN_1250939	8	18884287
131	4.235E-40	BAT5	NM_178592.3	ILMN_2731523	17	35237291
157	7.201E-40	CXADR		ILMN_2514377	16	78340630
225	9.37E-40	STX8	NM_018768.2	ILMN_3064056	11	68020355
196	2.134E-39	RPS15A	NM_170669.2	ILMN_2717621	7	125247949
221	6.782E-39	2810410P22RIK	NM_182994.1	ILMN_2692989	2	52254739
113	9.211E-39	PDXDC1	NM_053181.2	ILMN_2727980	16	13836112
181	1.017E-38	MBOAT2	NM_026037.2	ILMN_2782248	12	25548411
325	1.466E-38	ATP2C1	NM_175025.2	ILMN_2658392	9	105314001
49	2.232E-38	ZFP365	NM_178679.2	ILMN_2966034	10	67349464
30	3.574E-38	NUBP2	NM_011956.2	ILMN_2925567	17	25021151
131	3.97E-38	SSPN	NM_010656.2	ILMN_2741402	6	145913360
101	5.252E-38	ABHD1	NR_003522.1	ILMN_1245678	5	31257356
127	5.779E-38	MRPL35	NM_025430.2	ILMN_2753924	6	71765387
101	1.062E-37	ABHD1	NR_003522.1	ILMN_2672772	5	31255333
91	1.338E-37	ZFP68	NM_013844.2	ILMN_2455192	5	139047396
100	1.487E-37	ABHD1	NM_021304.2	ILMN_2672778	5	31229530
167	1.978E-37	CCDC88A	NM_176841.3	ILMN_1258136	11	29409085
245	2.581E-37	ANG	NM_007447.2	ILMN_2875251	14	51721418
5	2.639E-37	ACTL6B	NM_031404.4	ILMN_2696182	5	138010632
148	2.829E-37	ZFP367	NM_175494.4	ILMN_1257178	13	64235099
99	2.903E-37	MIPEP	NM_027436.2	ILMN_1222492	14	61522362
49	4.616E-37	THAP4	NM_025920.3	ILMN_2773540	1	95602011
88	5.316E-37	RBM45	NM_153405.2	ILMN_3163340	2	76218481
136	5.807E-37	SRPR	NM_026130.1	ILMN_2747480	9	35024334
205	6.992E-37	G430022H21RIK	NM_201638.1	ILMN_2818206	3	123071380
72	1.158E-36	DCP1B	NM_001033379.1	ILMN_3062378	6	119185732
83	1.248E-36	PRCP	NM_028243.2	ILMN_2639155	7	100082354
280	1.312E-36	PARP2	NM_009632.2	ILMN_2598576	14	51440489
78	1.778E-36	ZFP27	NM_001037707.1	ILMN_2490271	7	30679068
183	2.588E-36	FGFR1OP2	NM_026218.2	ILMN_2615468	6	146547446
193	2.951E-36	LCMT1	NM_025304.3	ILMN_2758728	7	130521566
66	3.838E-36	ZFP35	NM_011755.2	ILMN_2508595	18	24162927
144	4.788E-36	PHLDA3	NM_013750.1	ILMN_2628567	1	137665204
286	8.025E-36	RAD23A	NM_009010.4	ILMN_2662743	8	87361612
102	9.518E-36	SERPINI1	NM_009250.1	ILMN_3161679	3	75445777
221	1.022E-35	2810410P22RIK	NM_182994.1	ILMN_2692986	2	52254734
13	1.424E-35	PYGB	NM_153781.1	ILMN_2747754	2	150657139
53	1.506E-35	DMWD	NM_010058.1	ILMN_2711355	7	19667937
221	1.681E-35	ARL5A	NM_182994.2	ILMN_1221102	2	52254728

Table S3. Top 100 *cis* eQTLs, striatum

markerCount	minP	ILMN_Gene	RefSeq_ID	Probe_Id	chromosome	chrStart
273	9.4314E-98	PSMB6	NM_008946.2	ILMN_2895862	11	70343427
58	6.5077E-88	COPS8	NM_133805.3	ILMN_1218868	1	92509380
101	1.3833E-79	B3GALT6	NM_080445.4	ILMN_2687140	4	155364722
149	1.0523E-72	CASP9	NM_015733.4	ILMN_1217061	4	141371743
263	1.7681E-66	PCOLN3	NM_145606.1	ILMN_2847502	8	126090554
75	2.3952E-65	LRRC57	NM_025657.2	ILMN_2898944	2	120430648
107	5.0096E-65	MPV17L	NM_033564.1	ILMN_2794258	16	13862714
72	1.6426E-64	RUSC2	NM_199057.2	ILMN_3159149	4	43439821
283	4.3962E-64	UAP1	NM_133806.4	ILMN_2502542	1	172072552
77	1.1024E-62	TESK1	NM_011571.2	ILMN_1231930	4	43460830
68	2.2434E-62	RUSC2	NM_199057.2	ILMN_1214953	4	43439818
185	6.1986E-62	MRPL10	NM_026154.1	ILMN_2822622	11	96910086
140	9.4641E-60	IL17D	NM_145837.1	ILMN_2792502	14	58161838
256	4.8758E-59	RBM13	NM_026453.1	ILMN_3009910	8	32625759
35	4.0766E-58	PDRG1	NM_178939.2	ILMN_1249417	2	152834935
297	9.6992E-56	STARD7	NM_139308.1	ILMN_2676066	2	127124017
188	7.2142E-55	TTC27	NM_152817.3	ILMN_1238733	17	75262828
365	8.683E-55	ZFP330	NM_145600.1	ILMN_2825109	8	85287669
143	3.4289E-54	BAT5	NM_178592.3	ILMN_2731523	17	35237291
287	1.0772E-53	ARL3	NM_019718.2	ILMN_1238801	19	46626113
274	6.6851E-53	PRDX2	NM_011563.2	ILMN_2638354	8	87864469
250	2.9152E-52	1200015F23RIK	NM_001033136.2	ILMN_3150536	2	118962936
264	1.8694E-51	NUDT6	NM_153561.2	ILMN_1253773	3	37310483
67	3.6636E-50	KRT12	NM_010661.2	ILMN_2865527	11	99277085
230	3.7995E-50	G430022H21RIK	NM_201638.1	ILMN_2818206	3	123071380
197	1.0717E-49	FGFR1OP2	NM_026218.2	ILMN_2615468	6	146547446
44	1.5391E-49	CCRN4L	NM_009834.1	ILMN_1255422	3	51055149
212	1.6442E-49	THUMPD1	NM_145585.1	ILMN_2722488	7	126858959
179	2.572E-49	MBOAT2	NM_001083341.1	ILMN_1229343	12	25644557
149	2.8082E-49	GPN2	NM_133884.1	ILMN_1214375	4	133147483
21	1.1476E-47	YIPF4	NM_026417.3	ILMN_1255657	17	74899545
64	1.3934E-47	PAIP1	NM_145457.3	ILMN_2752569	13	120236564
301	2.204E-47	FCER1G	NM_010185.2	ILMN_2748875	1	173159942
18	3.0585E-47	TTL	NM_027192.1	ILMN_2961165	2	128919424
273	3.8208E-47	RPS3	NM_012052.1	ILMN_2594103	7	106628416
274	4.7457E-47	PRDX2	NM_011563.2	ILMN_1242829	8	87864541
235	2.1061E-46	FAM20B	NM_145413.4	ILMN_1247603	1	158611230
244	7.422E-46	H2AFJ	NM_177688.2	ILMN_2994806	6	136773689
113	9.8215E-46	PDXDC1	NM_053181.2	ILMN_2727980	16	13836112
9	1.4847E-45	KIF3A	NM_008443.3	ILMN_2621148	11	53406708
149	3.2564E-45	ZNHIT3	NM_001005223.1	ILMN_2914957	11	84727546
336	7.0845E-45	RPL29	NM_009082.2	ILMN_2730005	9	106331900
21	1.5506E-44	IGSF11	NM_170599.2	ILMN_2965399	16	39027008

333	3.0483E-44	CCNDBP1	NM_010761.2	ILMN_1240178	2	120842190
177	5.4265E-44	SC4MOL	NM_025436.1	ILMN_2823778	8	67610701
231	6.2553E-44	D10627	NM_001013379.2	ILMN_2912111	8	71752786
138	3.921E-43	CSNRP3	NM_153409.3	ILMN_2744731	2	65861141
78	4.0541E-43	CCNG2	NM_007635.3	ILMN_2700233	5	93704893
253	4.2934E-43	GPR19	NM_008157.1	ILMN_1257801	6	134819305
112	6.0307E-43	1700123O20RIK	NM_021437.1	ILMN_2597332	14	55309002
19	1.1289E-42	GDPD3	NM_024228.2	ILMN_2893879	7	133914694
141	3.0612E-42	SSPN	NM_010656.2	ILMN_2741402	6	145913360
192	3.1403E-42	CCDC88A	NM_176841.3	ILMN_1258136	11	29409085
159	3.3269E-42	CXADR		ILMN_2514377	16	78340630
258	5.0024E-42	ATP2C1	NM_175025.2	ILMN_2658392	9	105314001
110	9.7831E-42	ENPP5	NM_032003.1	ILMN_1217118	17	44223312
170	1.1234E-41	MBOAT2	NM_026037.2	ILMN_2782248	12	25548411
99	5.665E-41	AGPAT5	NM_026792.3	ILMN_1250939	8	18884287
129	8.6951E-41	CSRP1	NM_007791.4	ILMN_1260378	1	137648559
88	1.0932E-40	RBM45	NM_153405.2	ILMN_3163340	2	76218481
46	1.5185E-40	NLN	NM_029447.1	ILMN_2786567	13	104813546
161	2.5501E-40	PICALM		ILMN_2769656	7	97357127
211	2.7754E-40	6330503K22RIK	NM_182995.1	ILMN_3006575	7	125880065
104	4.3809E-40	MXRA8	NM_024263.3	ILMN_2927638	4	155217737
112	5.4938E-40	PSMB5	NM_011186.1	ILMN_2613469	14	55235381
81	5.7827E-40	CCDC53	NM_026070.2	ILMN_1244642	10	87682035
127	7.124E-40	EMB	NM_010330.3	ILMN_1218799	13	118062736
127	1.3602E-39	MRPL35	NM_025430.2	ILMN_2753924	6	71765387
260	2.2924E-39	LOC100048020	XM_001479635.1	ILMN_2653881	4	137998107
123	2.859E-39	9030612M13RIK	NM_172458.3	ILMN_2729447	17	32910323
112	2.9754E-39	PTCD3	NM_027275.3	ILMN_1228040	6	71831116
236	6.1207E-39	EG381438	NM_198657.1	ILMN_2998738	3	37906223
90	6.5527E-39	KRAS	NM_021284.4	ILMN_2773211	6	145168631
246	7.6936E-39	RSF1	NM_001081267.1	ILMN_2921163	7	104810161
188	7.9873E-39	MPG	NM_010822.2	ILMN_2600747	11	32131580
21	1.0938E-38	ATXN1	NM_009124.4	ILMN_1254409	13	45650599
9	2.3689E-38	MRPS10	NM_183086.1	ILMN_2841840	17	47515340
136	4.6051E-38	SLC7A14	NM_172861.2	ILMN_2968123	3	31105424
87	6.0743E-38	MIPEP	NM_027436.2	ILMN_1222492	14	61522362
151	6.7218E-38	SNTA1	NM_009228.1	ILMN_2734142	2	154202191
324	7.8334E-38	SSBP4	NM_133772.1	ILMN_2900827	8	73121502
113	1.5775E-37	TRIM59	NM_025863.2	ILMN_2993661	3	68839458
251	1.9667E-37	ANG	NM_007447.2	ILMN_2875251	14	51721418
72	2.0771E-37	DCP1B	NM_001033379.1	ILMN_3062378	6	119185732
246	2.2607E-37	GPS2	NM_019726.3	ILMN_2730784	11	69728828
142	2.5603E-37	SLC7A14	NM_172861.2	ILMN_2719361	3	31105423
175	3.2054E-37	SKIV2L2	NM_028151.2	ILMN_1218617	13	113658076
42	4.2058E-37	H2-T10	NM_010395.5	ILMN_2894678	17	36257609
8	5.3096E-37	ATP6V1D	NM_023721.2	ILMN_2647048	12	79944135

281	7.1114E-37	PARP2	NM_009632.2	ILMN_2598576	14	51440489
80	7.4386E-37	PRCP	NM_028243.2	ILMN_2639155	7	100082354
190	7.6224E-37	ZBTB22	NM_020625.3	ILMN_1233129	17	34056177
127	7.6756E-37	MED23	NM_027347.2	ILMN_2860964	10	24633135
361	9.7309E-37	SAMD4	NM_001037221.1	ILMN_3128535	14	47721611
274	1.4881E-36	BC026585	XM_129546.2	ILMN_1221257	1	159418777
205	1.5641E-36	2310010B21RIK	NM_027123.2	ILMN_2696360	13	68730864
5	1.8409E-36	ACTL6B	NM_031404.4	ILMN_2696182	5	138010632
305	1.9959E-36	TMEM87A	NM_173734.2	ILMN_2969172	2	120181373
287	2.5049E-36	1200003I07RIK	NM_181274.3	ILMN_3135697	8	72229274
101	3.509E-36	ABHD1	NR_003522.1	ILMN_2672772	5	31255333

Table S4. Gene co-expression modules

	Hippocampus Module	Number of Genes		Striatum Module	Number of Genes
1	skyblue3	34	1	paleturquoise	34
2	yellowgreen	39	2	steelblue	43
3	sienna3	39	3	saddlebrown	46
4	darkmagenta	43	4	skyblue	50
5	darkolivegreen	44	5	darkorange	65
6	violet	60	6	white	65
7	paleturquoise	65	7	orange	72
8	steelblue	67	8	darkgreen	82
9	saddlebrown	73	9	darkred	90
10	skyblue	73	10	royalblue	93
11	white	76	11	lightcyan	128
12	orange	100	12	midnightblue	145
13	darkturquoise	128	13	cyan	163
14	darkgreen	131	14	greenyellow	339
15	darkred	154	15	magenta	360
16	royalblue	163	16	pink	430
17	grey60	184	17	grey60	488
18	cyan	229	18	black	528
19	greenyellow	414	19	darkgrey	621
20	magenta	469	20	green	730
**21**	**darkgrey**	**492**	21	darkturquoise	1031
22	black	520	22	brown	1275
23	lightyellow	604	23	blue	1408
24	lightcyan	696	24	red	2829
25	yellow	1120	25	grey	14582
26	green	1249			
27	blue	2592			
28	brown	2820			
29	turquoise	4574			
30	grey	8445			

Table S5. Functional classification for genes in Context Fear module 1 (CF1)

GOID	Ontology	Term	q	m	t	k	log_odds_ratio	p
GO:0005622	cellular_component	intracellular	1527	11917	25697	2820	0.223583745	1.54E-16
GO:0044424	cellular_component	intracellular part	1477	11581	25697	2820	0.216814772	1.31E-14
GO:0043229	cellular_component	intracellular organelle	1256	9759	25697	2820	0.229936015	3.55E-12
GO:0043226	cellular_component	organelle	1256	9769	25697	2820	0.228458449	5.02E-12
GO:0043231	cellular_component	intracellular membrane-bounded organelle	1128	8645	25697	2820	0.249733978	5.67E-12
GO:0043227	cellular_component	membrane-bounded organelle	1129	8657	25697	2820	0.2490112	6.35E-12
GO:0009987	biological_process	cellular process	1347	10821	25697	2820	0.181820801	1.10E-08
GO:0044237	biological_process	cellular metabolic process	909	6995	25697	2820	0.243861016	3.39E-08
GO:0005737	cellular_component	cytoplasm	998	7780	25697	2820	0.225174439	4.87E-08
GO:0044444	cellular_component	cytoplasmic part	606	4483	25697	2820	0.300758073	3.69E-07
GO:0005739	cellular_component	mitochondrion	203	1283	25697	2820	0.527863336	4.38E-06
GO:0044260	biological_process	cellular macromolecule metabolic process	715	5494	25697	2820	0.24599111	6.56E-06
GO:0044238	biological_process	primary metabolic process	925	7356	25697	2820	0.196436665	2.20E-05
GO:0008152	biological_process	metabolic process	1042	8392	25697	2820	0.178173474	2.62E-05
GO:0005634	cellular_component	nucleus	692	5350	25697	2820	0.237137925	3.16E-05
GO:0046983	molecular_function	protein dimerization activity	62	307	25697	2820	0.879934339	0.000123
GO:0031967	cellular_component	organelle envelope	108	631	25697	2820	0.641224181	0.0001842
GO:0031975	cellular_component	envelope	108	633	25697	2820	0.636658687	0.000214
GO:0000166	molecular_function	nucleotide binding	353	2585	25697	2820	0.315408682	0.000581
GO:0017076	molecular_function	purine nucleotide binding	312	2256	25697	2820	0.33368374	0.0007735
GO:0032553	molecular_function	ribonucleotide binding	299	2156	25697	2820	0.337693085	0.000974
GO:0032555	molecular_function	purine ribonucleotide binding	299	2156	25697	2820	0.337693085	0.000974
GO:0043170	biological_process	macromolecule metabolic process	764	6116	25697	2820	0.186889011	0.0013494
GO:0044446	cellular_component	intracellular organelle part	393	2948	25697	2820	0.280697567	0.0016177
GO:0044267	biological_process	cellular protein metabolic process	352	2607	25697	2820	0.299089625	0.0016299
GO:0005635	cellular_component	nuclear envelope	35	156	25697	2820	1.031713672	0.0019521
GO:0044422	cellular_component	organelle part	401	3028	25697	2820	0.27114181	0.0024112
GO:0018346	biological_process	protein amino acid prenylation	4	5	25697	2820	2.865904779	0.0026906
GO:0033036	biological_process	macromolecule localization	163	1092	25697	2820	0.443803887	0.002925
GO:0008104	biological_process	protein localization	141	928	25697	2820	0.469403231	0.0042184
GO:0006739	biological_process	NADP metabolic process	9	21	25697	2820	1.965440453	0.004692
GO:0030163	biological_process	protein catabolic process	113	717	25697	2820	0.522182528	0.005085
GO:0003824	molecular_function	catalytic activity	806	6551	25697	2820	0.164969469	0.0059773
GO:0042802	molecular_function	identical protein binding	48	249	25697	2820	0.812793443	0.0059773

GO:0051603	biological_process	proteolysis involved in cellular protein catabolic process	108	684	25697	2820	0.524867861	0.0067623
GO:0005525	molecular_function	GTP binding	75	441	25697	2820	0.632016719	0.0075668
GO:0030529	cellular_component	ribonucleoprotein complex	85	515	25697	2820	0.588795188	0.0080469
GO:0044257	biological_process	cellular protein catabolic process	108	687	25697	2820	0.518554087	0.0080469
GO:0009056	biological_process	catabolic process	181	1257	25697	2820	0.391909827	0.008325
GO:0019001	molecular_function	guanyl nucleotide binding	76	450	25697	2820	0.621979196	0.0084909
GO:0032561	molecular_function	guanyl ribonucleotide binding	76	450	25697	2820	0.621979196	0.0084909
GO:0016740	molecular_function	transferase activity	310	2320	25697	2820	0.284048189	0.0117608
GO:0010467	biological_process	gene expression	388	2976	25697	2820	0.248586905	0.0124876
GO:0015031	biological_process	protein transport	125	826	25697	2820	0.463619187	0.0129201
GO:0045184	biological_process	establishment of protein localization	125	827	25697	2820	0.461873639	0.0135731
GO:0019321	biological_process	pentose metabolic process	4	6	25697	2820	2.602870373	0.0135731
GO:0018342	biological_process	protein prenylation	4	6	25697	2820	2.602870373	0.0135731
GO:0004308	molecular_function	exo-alpha-sialidase activity	4	6	25697	2820	2.602870373	0.0135731
GO:0016997	molecular_function	alpha-sialidase activity	4	6	25697	2820	2.602870373	0.0135731
GO:0032991	cellular_component	macromolecular complex	344	2615	25697	2820	0.261502397	0.0159775
GO:0008318	molecular_function	protein prenyltransferase activity	5	9	25697	2820	2.339835967	0.0169367
GO:0016772	molecular_function	transferase activity, transferring phosphorus-containing groups	187	1323	25697	2820	0.365129987	0.0170969
GO:0044265	biological_process	cellular macromolecule catabolic process	114	749	25697	2820	0.47190098	0.0192849
GO:0019941	biological_process	modification-dependent protein catabolic process	104	675	25697	2820	0.4895289	0.0220008
GO:0043632	biological_process	modification-dependent macromolecule catabolic process	104	675	25697	2820	0.4895289	0.0220008
GO:0044248	biological_process	cellular catabolic process	149	1026	25697	2820	0.404186379	0.0220008
GO:0009057	biological_process	macromolecule catabolic process	121	810	25697	2820	0.444918013	0.0266769
GO:0016070	biological_process	RNA metabolic process	109	717	25697	2820	0.47018789	0.0266769
GO:0006511	biological_process	ubiquitin-dependent protein catabolic process	103	671	25697	2820	0.484164445	0.0268211
GO:0008408	molecular_function	3'-5' exonuclease activity	7	17	25697	2820	1.907724955	0.0300059
GO:0016884	molecular_function	carbon-nitrogen ligase activity, with glutamine as amido-N-donor	7	17	25697	2820	1.907724955	0.0300059
GO:0012505	cellular_component	endomembrane system	77	478	25697	2820	0.553752607	0.0336492
GO:0008270	molecular_function	zinc ion binding	316	2409	25697	2820	0.257394944	0.0352466
GO:0051766	molecular_function	inositol trisphosphate kinase activity	5	10	25697	2820	2.187832874	0.0360964
GO:0034641	biological_process	cellular nitrogen compound metabolic process	456	3600	25697	2820	0.206941697	0.0372876
GO:0006396	biological_process	RNA processing	80	503	25697	2820	0.535346379	0.0391974
GO:0070584	biological_process	mitochondrion morphogenesis	4	7	25697	2820	2.380477952	0.0397828
GO:0006012	biological_process	galactose metabolic process	4	7	25697	2820	2.380477952	0.0397828
GO:0044428	cellular_component	nuclear part	160	1130	25697	2820	0.367653911	0.0427709
GO:0010181	molecular_function	FMN binding	8	22	25697	2820	1.728401255	0.0427709

Table S6. Functional classification for genes in Context Fear module 1 (CF2)

GOID	Ontology	Term	q	m	log_odds_ratio	p
GO:0005622	cellular_component	intracellular	298	11917	0.385232861	2.33E-08
GO:0044424	cellular_component	intracellular part	289	11581	0.382251286	1.05E-07
GO:0005737	cellular_component	cytoplasm	211	7777	0.502898981	2.87E-07
GO:0044444	cellular_component	cytoplasmic part	131	4475	0.61254885	3.12E-05
GO:0044446	cellular_component	intracellular organelle part	94	2948	0.735865858	6.97E-05
GO:0044422	cellular_component	organelle part	94	2974	0.723197735	0.000102252
GO:0005791	cellular_component	rough endoplasmic reticulum	3	5	4.969832222	0.000189871
GO:0030867	cellular_component	rough endoplasmic reticulum membrane	3	5	4.969832222	0.000189871
GO:0031967	cellular_component	organelle envelope	31	641	1.33681358	0.000275161
GO:0031975	cellular_component	envelope	31	643	1.332319199	0.000289428
GO:0043231	cellular_component	intracellular membrane-bounded organelle	215	8645	0.377340417	0.000317968
GO:0043227	cellular_component	membrane-bounded organelle	215	8657	0.375339221	0.000349763
GO:0043229	cellular_component	intracellular organelle	236	9727	0.341661663	0.000427685
GO:0043226	cellular_component	organelle	236	9741	0.339586695	0.000476649
GO:0016833	molecular_function	oxo-acid-lyase activity	3	6	4.706797816	0.000502354
GO:0031090	cellular_component	organelle membrane	35	816	1.163655491	0.000907501
GO:0065002	biological_process	intracellular protein transmembrane transport	9	89	2.400989386	0.00197888
GO:0044429	cellular_component	mitochondrial part	26	547	1.311840511	0.002057787
GO:0016607	cellular_component	nuclear speck	4	16	3.706797816	0.002238435
GO:0019866	cellular_component	organelle inner membrane	21	398	1.462490618	0.002444955
GO:0008104	biological_process	protein localization	37	928	1.058270186	0.002466883
GO:0015031	biological_process	protein transport	34	826	1.104262686	0.002675773
GO:0045184	biological_process	establishment of protein localization	34	827	1.102517138	0.002708913
GO:0043021	molecular_function	ribonucleoprotein binding	5	29	3.170744916	0.003532873
GO:0005813	cellular_component	centrosome	7	59	2.631509689	0.003588551
GO:0005743	cellular_component	mitochondrial inner membrane	20	380	1.458870302	0.003591612
GO:0005740	cellular_component	mitochondrial envelope	23	479	1.326477926	0.004532591
GO:0005739	cellular_component	mitochondrion	45	1283	0.873345457	0.007670555
GO:0031966	cellular_component	mitochondrial membrane	22	466	1.30204329	0.008161473

GO:0043022	molecular_function	ribosome binding	3	11	3.832328698	0.008166989
GO:0055085	biological_process	transmembrane transport	10	131	1.995302909	0.009356374
GO:0006890	biological_process	retrograde vesicle-mediated transport, Golgi to ER	2	5	4.384869721	0.013577213
GO:0060180	biological_process	female mating behavior	2	5	4.384869721	0.013577213
GO:0033036	biological_process	macromolecule localization	39	1092	0.899442894	0.014821171
GO:0012505	cellular_component	endomembrane system	22	488	1.235492097	0.015313506
GO:0033057	biological_process	reproductive behavior in a multicellular organism	3	13	3.591320598	0.015502907
GO:0007007	biological_process	inner mitochondrial membrane organization	3	13	3.591320598	0.015502907
GO:0045039	biological_process	protein import into mitochondrial inner membrane	3	13	3.591320598	0.015502907
GO:0042719	cellular_component	mitochondrial intermembrane space protein transporter complex	3	13	3.591320598	0.015502907
GO:0005815	cellular_component	microtubule organizing center	7	77	2.247366197	0.02049463
GO:0007617	biological_process	mating behavior	3	14	3.484405394	0.02049463
GO:0006626	biological_process	protein targeting to mitochondrion	3	14	3.484405394	0.02049463
GO:0070585	biological_process	protein localization in mitochondrion	3	14	3.484405394	0.02049463
GO:0006166	biological_process	purine ribonucleoside salvage	2	6	4.121835315	0.02239209
GO:0043101	biological_process	purine salvage	2	6	4.121835315	0.02239209
GO:0043174	biological_process	nucleoside salvage	2	6	4.121835315	0.02239209
GO:0060491	biological_process	regulation of cell projection assembly	2	6	4.121835315	0.02239209
GO:0043015	molecular_function	gamma-tubulin binding	2	6	4.121835315	0.02239209
GO:0005093	molecular_function	Rab GDP-dissociation inhibitor activity	2	6	4.121835315	0.02239209
GO:0009103	biological_process	lipopolysaccharide biosynthetic process	2	6	4.121835315	0.02239209
GO:0016830	molecular_function	carbon-carbon lyase activity	5	45	2.536872814	0.034289251
GO:0046479	biological_process	glycosphingolipid catabolic process	2	7	3.899442894	0.037545359
GO:0008653	biological_process	lipopolysaccharide metabolic process	2	7	3.899442894	0.037545359
GO:0019377	biological_process	glycolipid catabolic process	2	8	3.706797816	0.043963871
GO:0004427	molecular_function	inorganic diphosphatase activity	2	8	3.706797816	0.043963871
GO:0006612	biological_process	protein targeting to membrane	4	33	2.662403696	0.045860944
GO:0034613	biological_process	cellular protein localization	19	444	1.160309463	0.047858188
GO:0044450	cellular_component	microtubule organizing center part	3	19	3.043832803	0.047882564
GO:0019098	biological_process	reproductive behavior	3	19	3.043832803	0.047882564
GO:0005783	cellular_component	endoplasmic reticulum	35	1031	0.826252215	0.049063572
GO:0070727	biological_process	cellular macromolecule localization	19	446	1.153825429	0.049317738

Figure S1. Cluster dendrogram by behavioral phenotype across HMDP

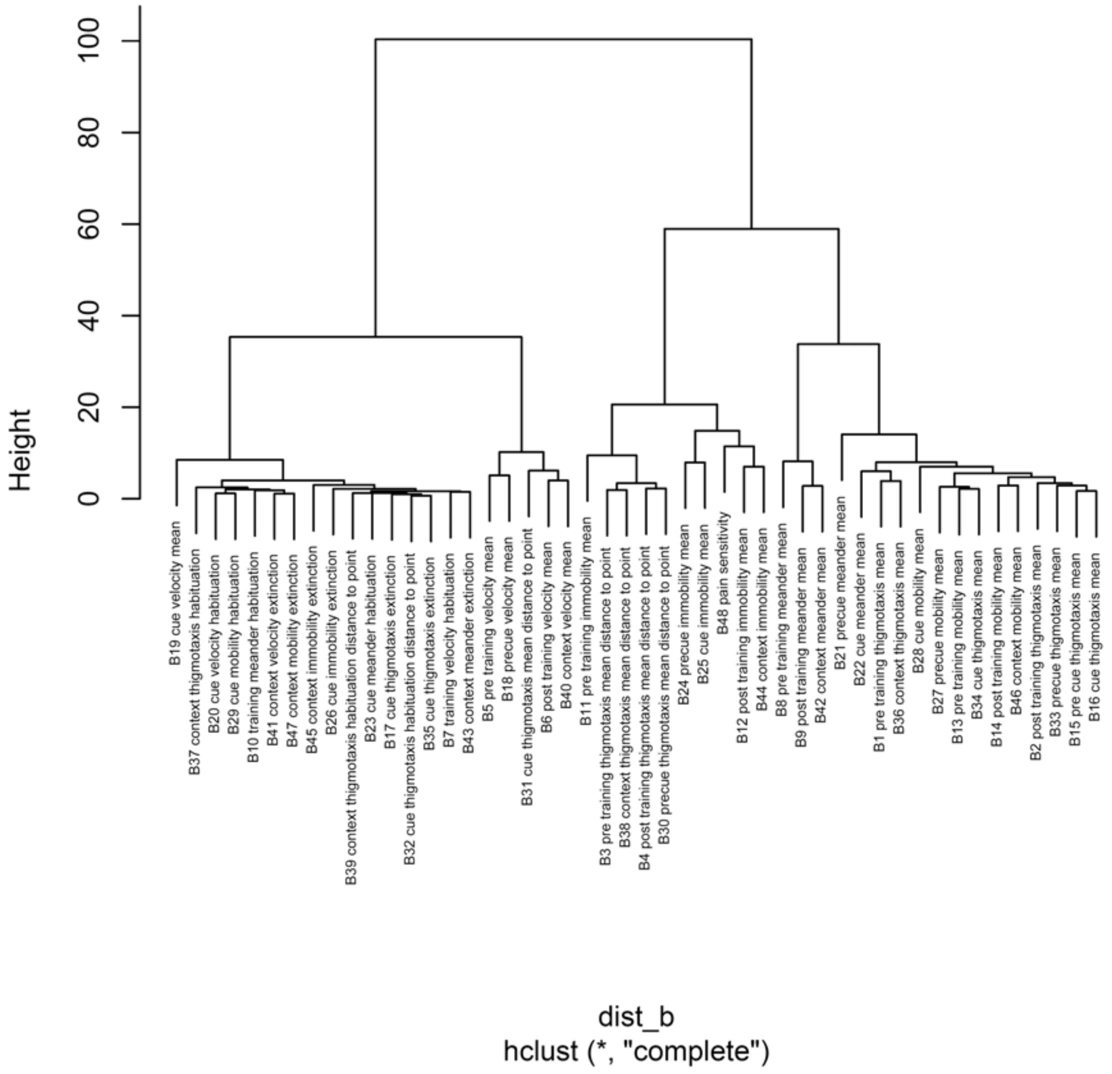

Figure S2. Mapped locus for B25 cue immobility mean on chromosome 7

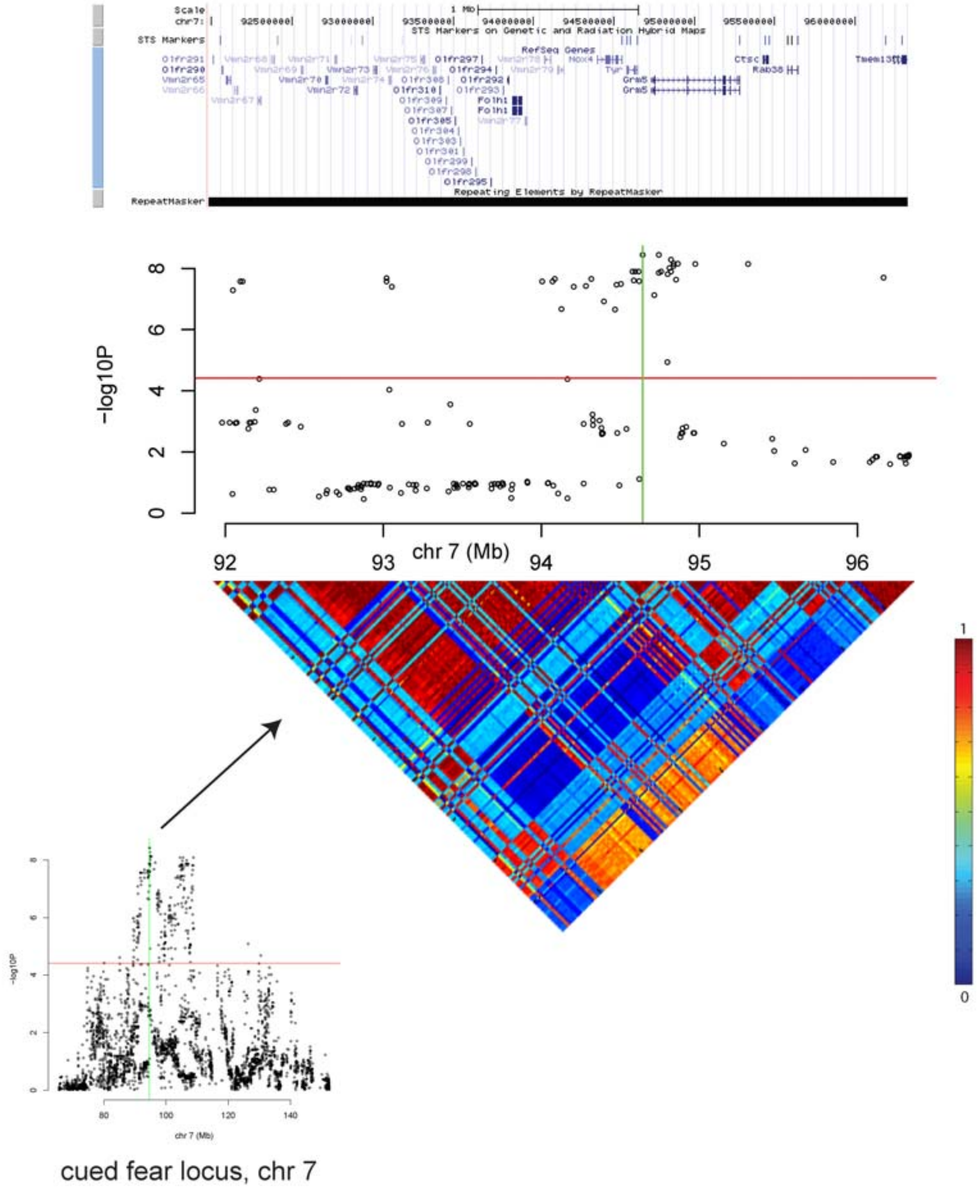

Figure S3. QTL plots for 48 tested behavioral phenotypes after EMMA population structure correction. The red line denotes genome-wide significance threshold of $P = 4.1 \times 10^{-6}$, equivalent to FDR < 0.05.

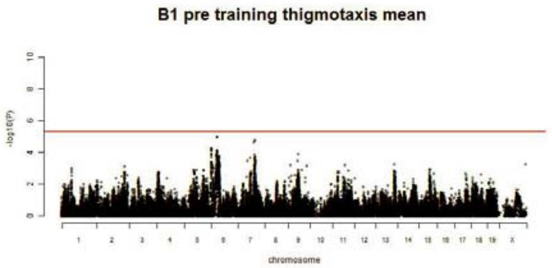

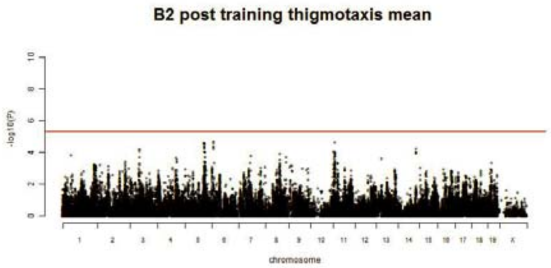

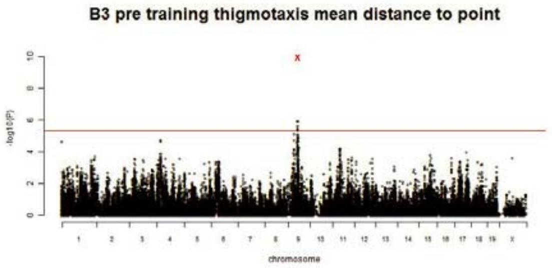

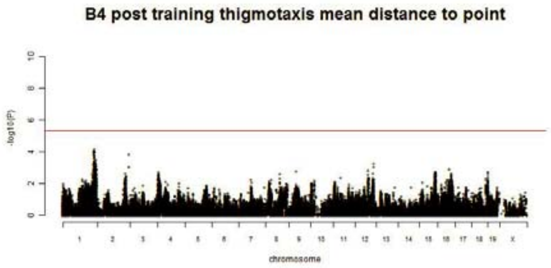

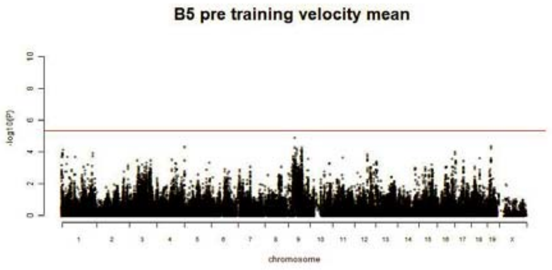

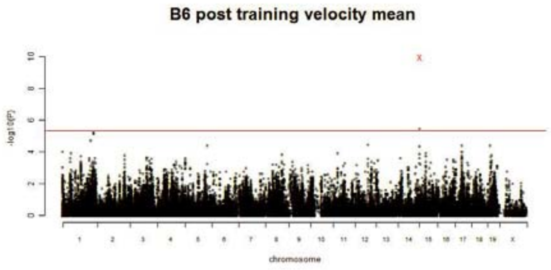

B7 training velocity habituation

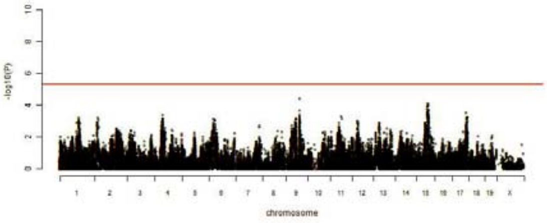

B8 pre training meander mean

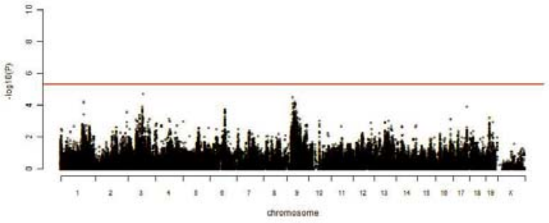

B9 post training meander mean

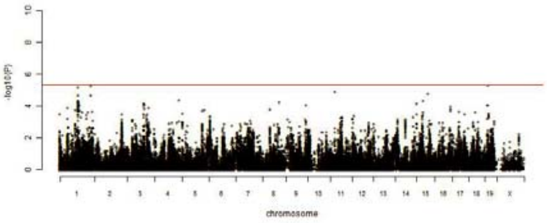

B10 training meander habituation

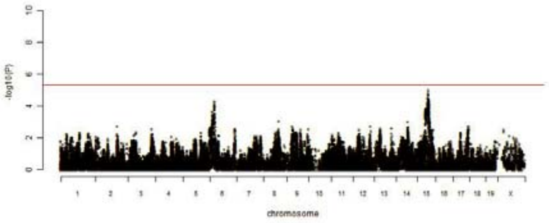

B11 pre training immobility mean

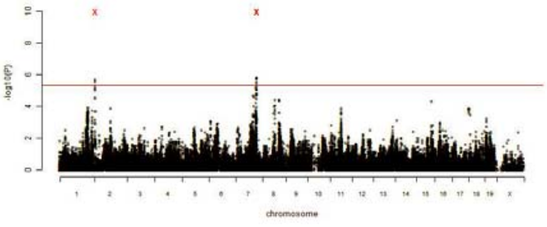

B12 post training immobility mean

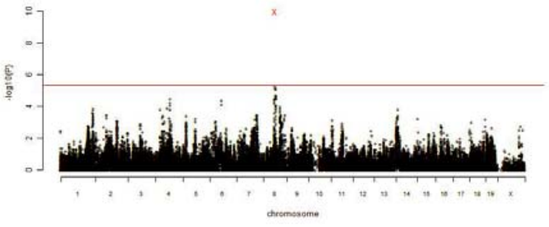

B13 pre training mobility mean

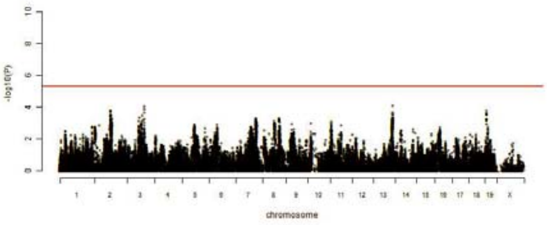

B14 post training mobility mean

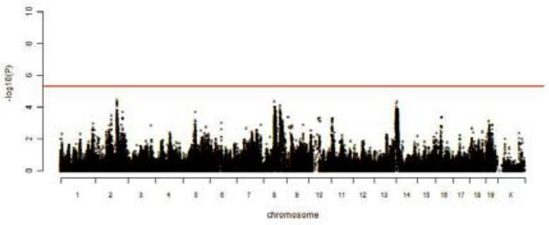

B15 pre cue thigmotaxis mean

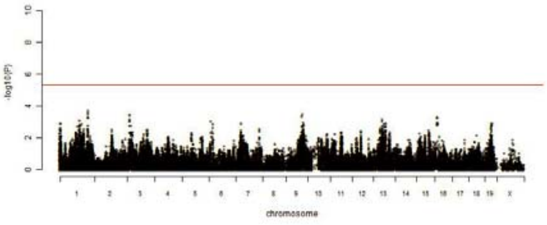

B16 cue thigmotaxis mean

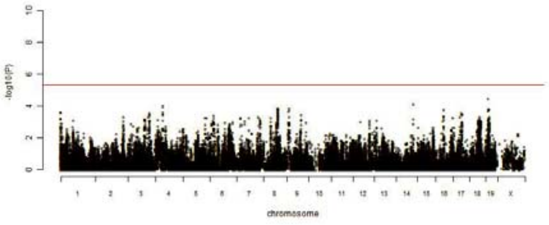

B17 cue thigmotaxis extinction

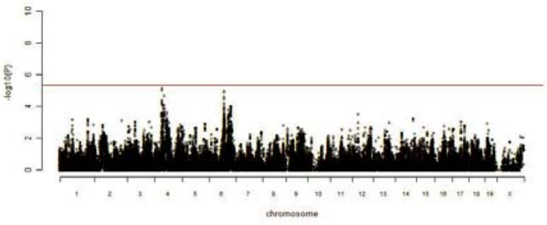

B18 pre cue velocity mean

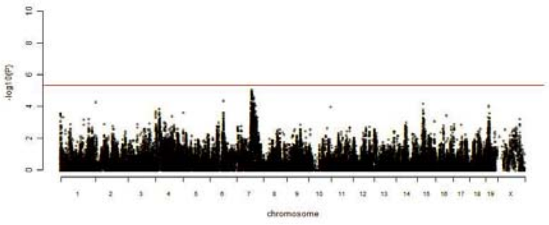

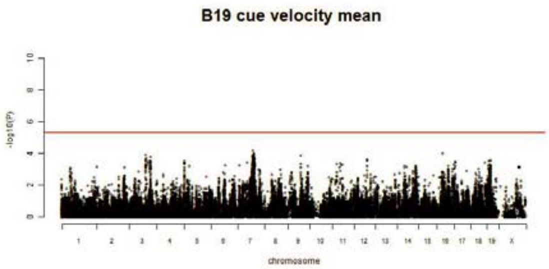
B19 cue velocity mean
-log10(P)
chromosome

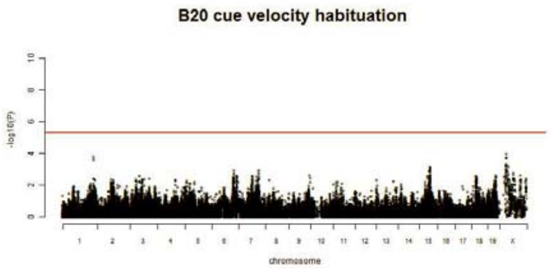
B20 cue velocity habituation
-log10(P)
chromosome

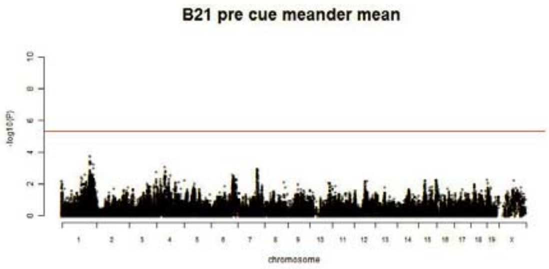
B21 pre cue meander mean
-log10(P)
chromosome

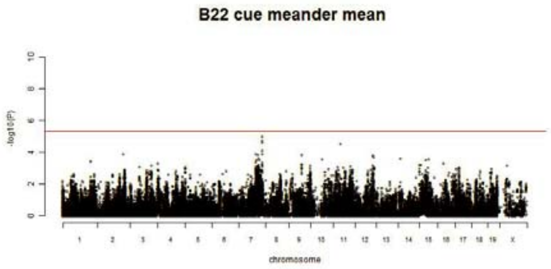
B22 cue meander mean
-log10(P)
chromosome

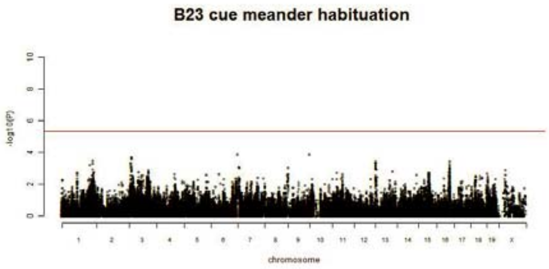
B23 cue meander habituation
-log10(P)
chromosome

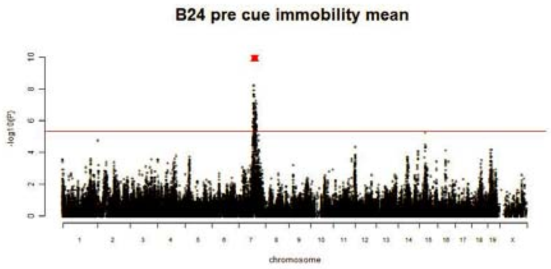
B24 pre cue immobility mean
-log10(P)
chromosome

B25 cue immobility mean

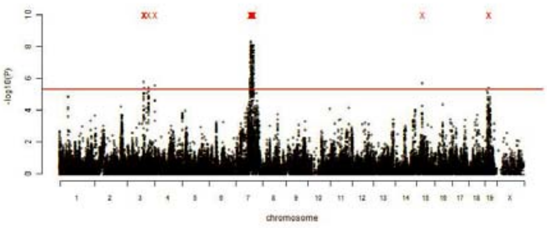

B26 cue immobility extinction

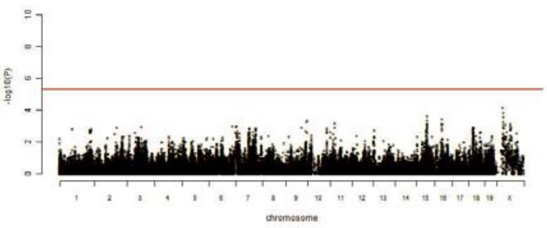

B27 pre cue mobility mean

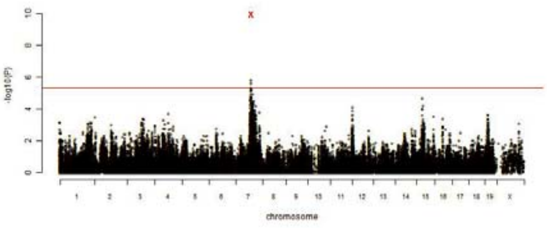

B28 cue mobility mean

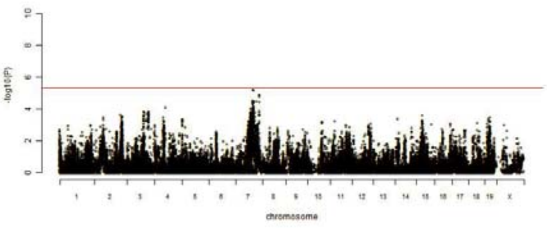

B29 cue mobility habituation

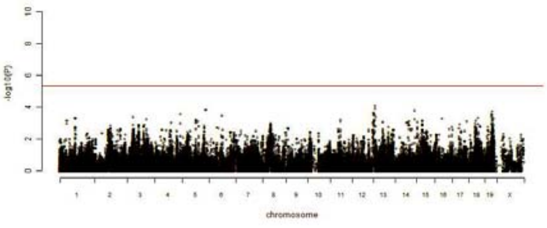

B30 cue thigmotaxis mean distance to point

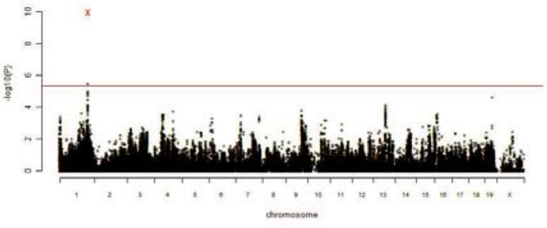

B31 cue thigmotaxis mean distance to point

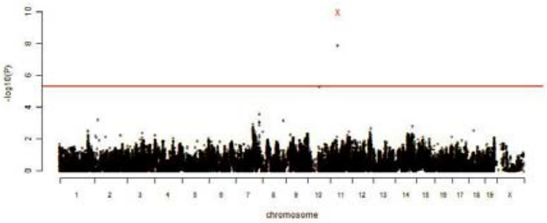

B32 cue thigmotaxis habituation distance to point

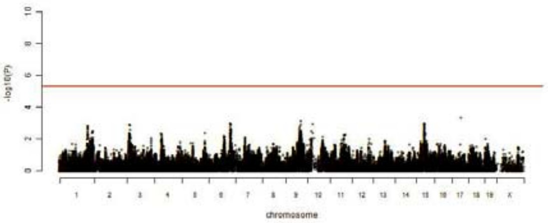

B33 cue thigmotaxis mean

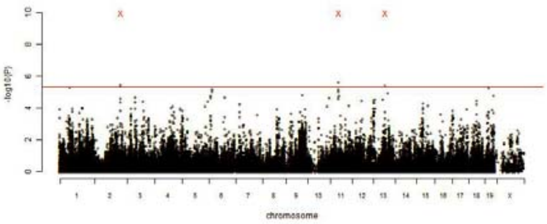

B34 cue thigmotaxis mean

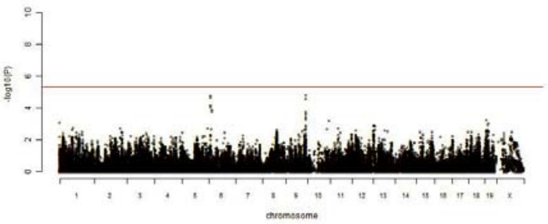

B35 cue thigmotaxis extinction

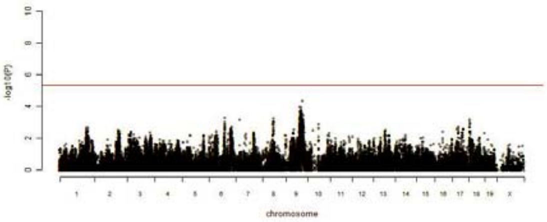

B36 context thigmotaxis mean

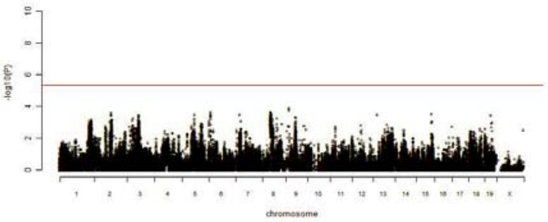

B37 context thigmotaxis habituation

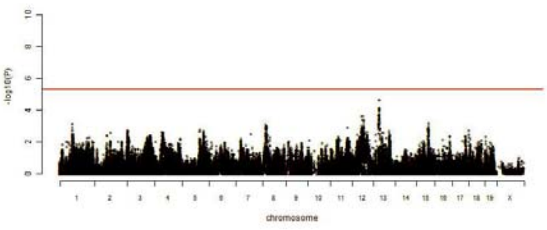

B38 context thigmotaxis mean distance to point

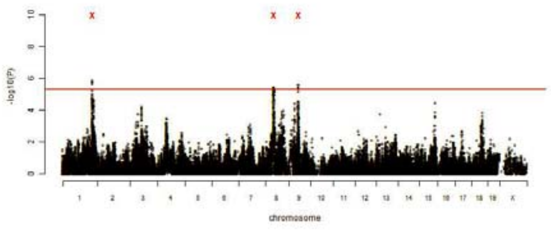

B39 context thigmotaxis habituation distance to point

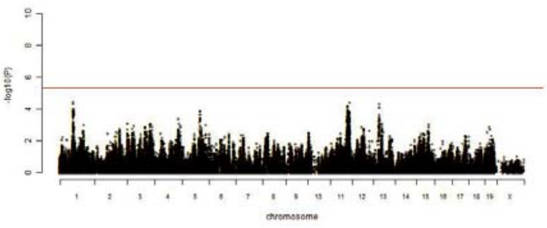

B40 context velocity mean

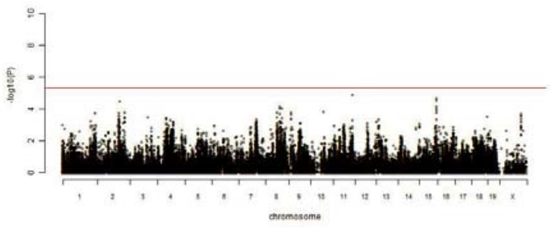

B41 context velocity extinction

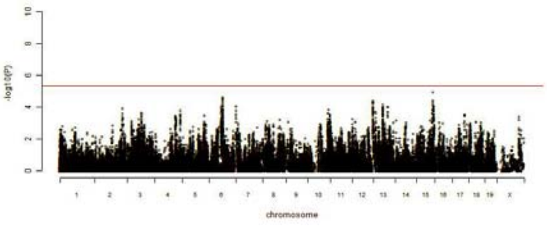

B42 context meander mean

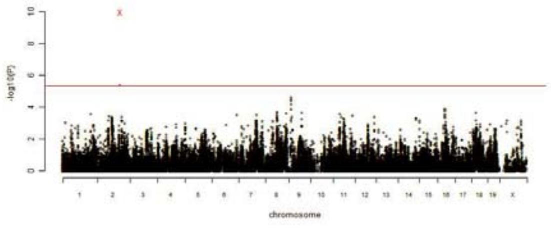

B43 context meander extinction

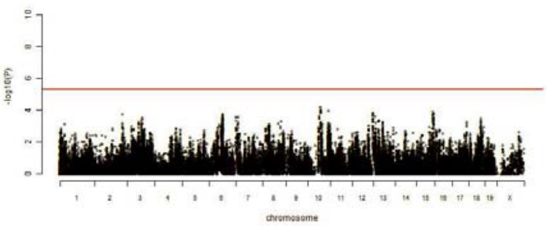

B44 context immobility mean

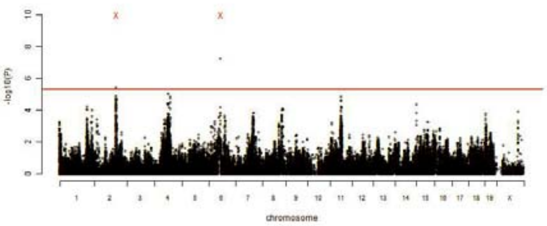

B45 context immobility extinction

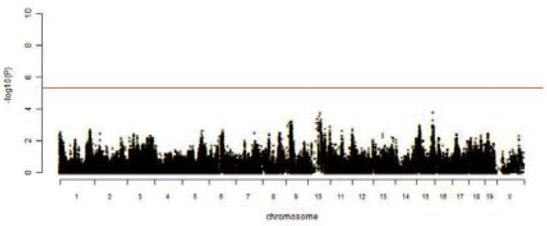

B46 context mobility mean

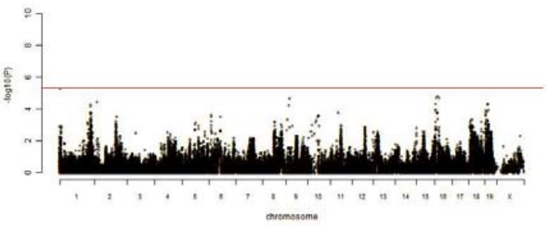

B47 context mobility extinction

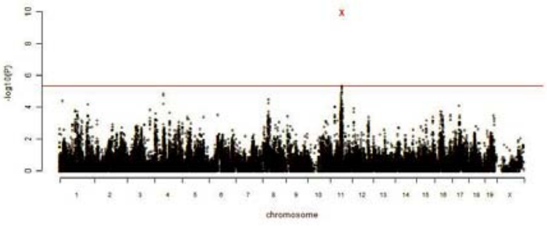

B48 pain sensitivity

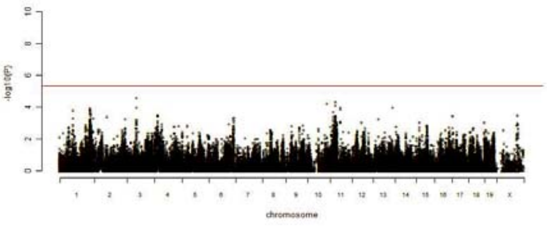

Figure S4. Hippocampus eQTLs. Genome location of SNP marker shown on the X axis, while genome probe start position is denoted by the Y axis. The X marginal plot represents number of probes regulated by each marker and shows hotspots of gene regulation. The Y marginal plot denotes number of markers regulating each probe. Each point represents a *P* value surpassing the genome wide significance threshold $P < 9.21 \times 10^{-6}$, or FDR < 5%.

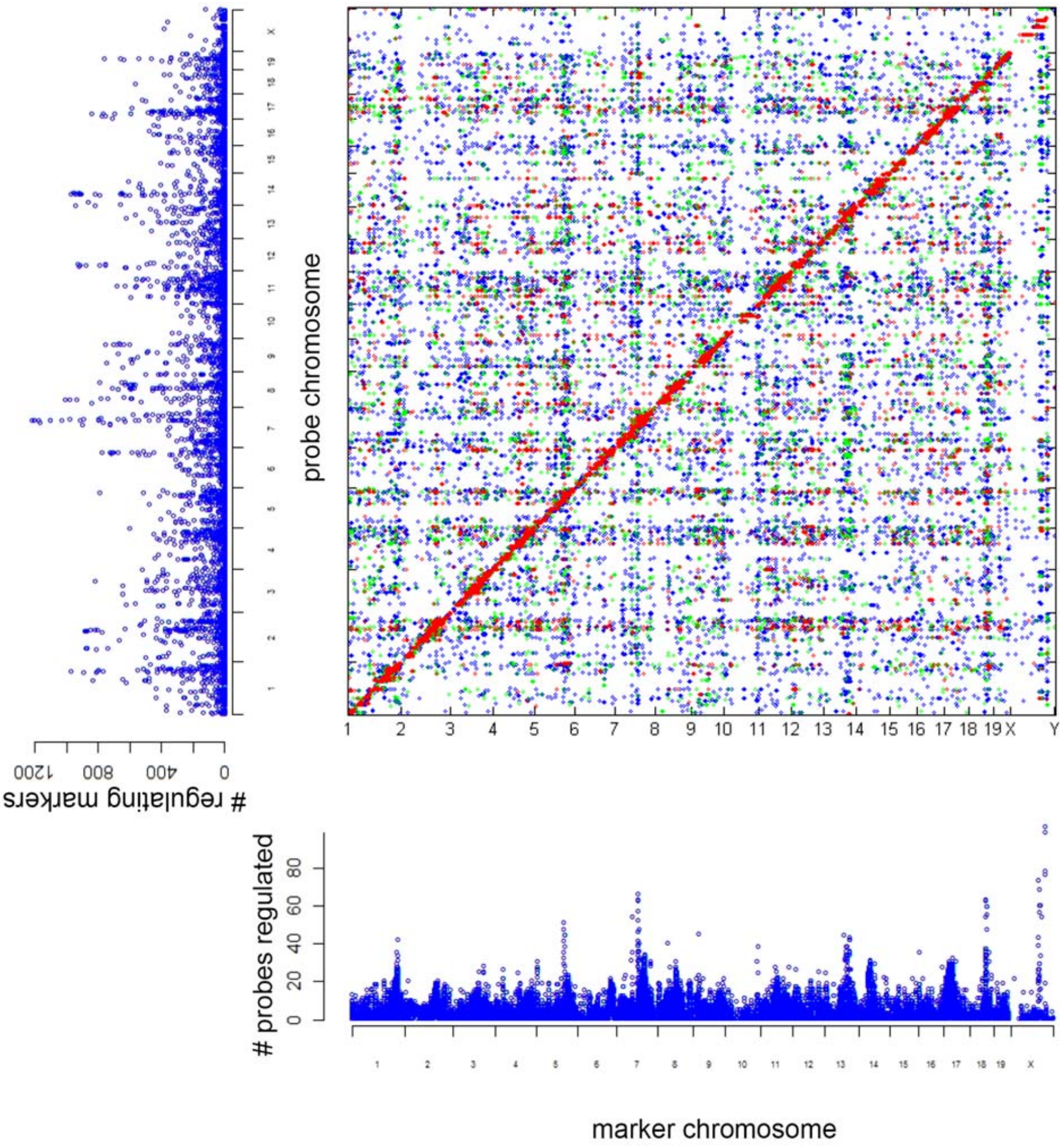

Figure S5. Striatum eQTLs. Genome location of the SNP marker shown on X axis, while genome probe start position is shown on Y. The X marginal plot represents number of probes regulated by each marker and shows hotspots of gene regulation. The Y marginal plot denotes number of markers regulating each probe. Each point represents a *P* value surpassing the genome wide significance threshold $P < 1.19 \times 10^{-5}$ or FDR < 5%.

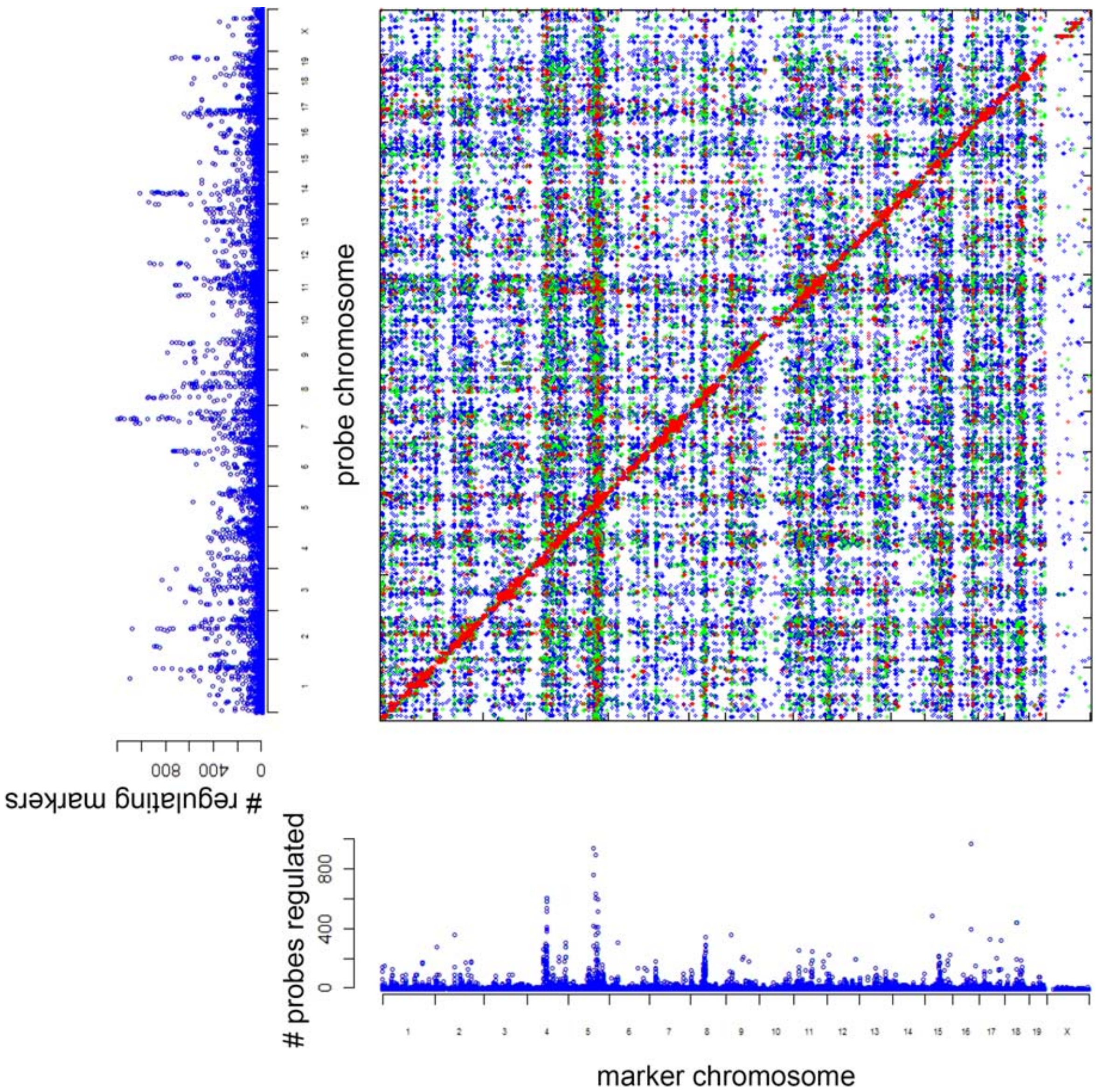

Figure S6. Hippocampus Module-trait correlations

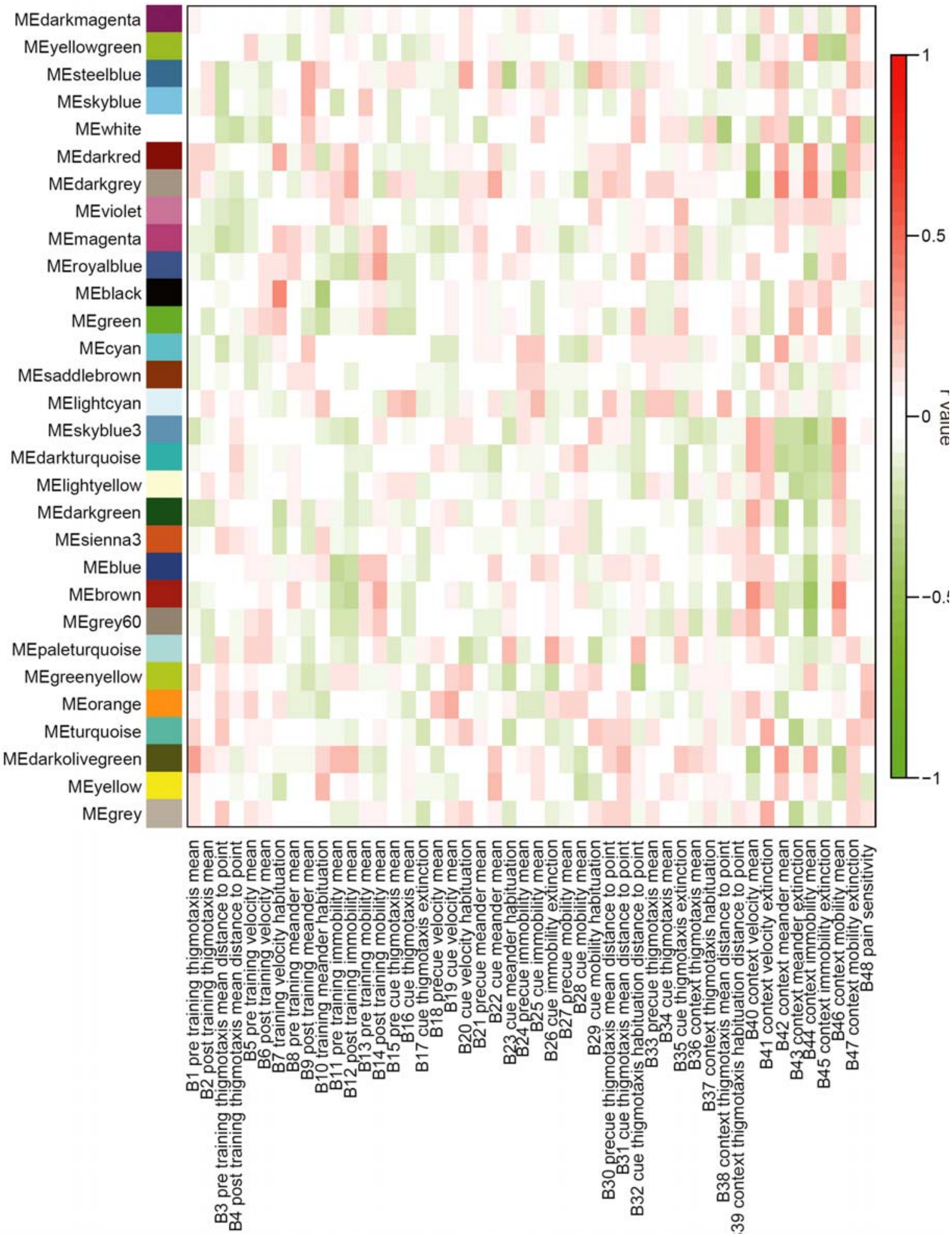

Behavioral phenotypes (1 – 48)

Figure S7. Striatum Module-trait correlations

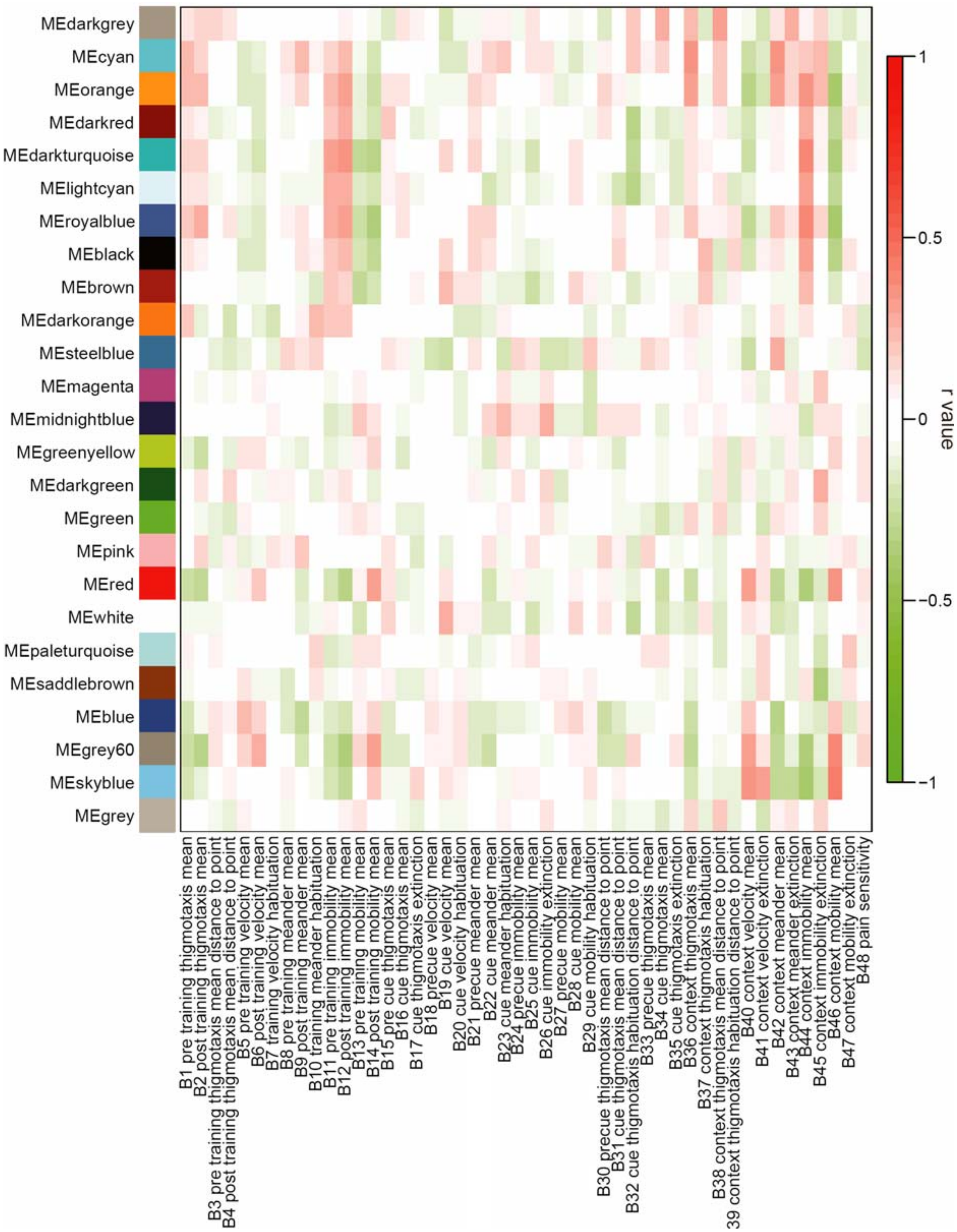

Behavioral phenotypes (1 – 48)

Figure S8. Striatum NEO results

SNP Marker Position (*Mbp*)	chr7:125.087101	chr7:94.799195	chr8:73.928238
RefSeq Gene	*6330503K22RIK*	*Kif3a*	*Plvap*
Probe Position (*Mbp*)	chr7:125.880065	chr11:53.406708	chr8:74.021817
Gene Expression QTL ($-\log_{10}P$)	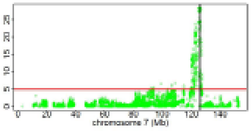	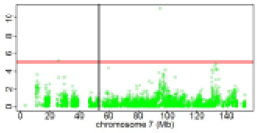	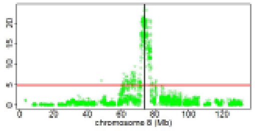
Behavioral QTL ($-\log_{10}P$)	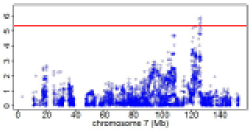	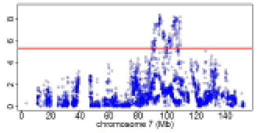	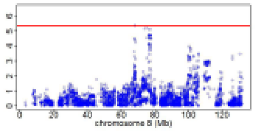
Quantitative Behavioral Phenotype	B11: Pre training immobility mean	B25: Cue immobility mean	B12: Post training immobility mean
LEO.NB.AtoB	2	1.21	0.662
RMSEA	0	0	0
